# Bridging phenotype and function in bladder cancer using immuno-competent organoids and *ex vivo* drug screening

**DOI:** 10.1186/s13046-026-03701-x

**Published:** 2026-04-01

**Authors:** Carlotta Frascolla, Riccardo Mastroianni, Catarina Macedo-Silva, Marta De Menna, Giulia Orlandi, Daniela Angela Covino, Martina Minoli, Martina Radic, Federico La Manna, Claudio Pulito, Sebastiano Vaccarella, Frauke Goeman, Ludovica Ciuffreda, Matteo Allegretti, Roland Seiler, Bernhard Kiss, Valentina De Pascale, Federica Runci, Fabio Calabrò, Yaron Vinik, Sima Lev, Sabrina Strano, Maurizio Fanciulli, Brindusa Ana Maria Arteni, Simona Di Martino, Andrea Russo, Sara Donzelli, Marianna Kruithof-de Julio, Giovanni Blandino, Giuseppe Simone

**Affiliations:** 1 Translational Oncology Research Unit, IRCCS Regina Elena National Cancer Institute, Rome, Italy; 2 Department of Sciences, Section of Biomedical Sciences and Technologies, Roma Tre University, Rome, Italy; 3 Uro-Oncology Program, IRCCS Regina Elena National Cancer Institute, Rome, Italy; 4 Department of Urology, IRCCS Regina Elena National Cancer Institute, Rome, Italy; 5 Department for BioMedical Research (DBMR), Urology Research Laboratory, University of Bern, Bern, Switzerland; 6 Department for BioMedical Research, Translational Organoid Research, University of Bern, Bern, Switzerland; 7 Microbiology and Virology Unit, San Gallicano Dermatological Institute IRCCS, Rome, Italy; 8 Gene Expression and Cancer Models Unit, Department of Research and Advanced Technologies Translational Research Area, IRCCS Regina Elena National Cancer Institute, Rome, Italy; 9 Department of Urology, University Hospital Bern, Inselspital, Bern, Switzerland; 10 Department of Urologie, Spitalzentrum, Biel, Switzerland; 11 Medical Oncology 1, IRCCS Regina Elena National Cancer Institute, Rome, Italy; 12 Molecular Cell Biology Department, Weizmann Institute of Science, Rehovot, Israel; 13 Pathology Unit, Tissue Biobank IRCCS Regina Elena National Cancer Institute, Rome, Italy; 14 Pathology Unit, IRCCS Regina Elena National Cancer Institute, Rome, Italy

**Keywords:** Bladder cancer, Patient-derived organoids (PDOs), Tumor microenvironment (TME), Immune preservation, Immunotherapy, Spatial heterogeneity, Target therapy, Precision oncology

## Abstract

**Background:**

Bladder cancer (BLC) remains a clinically challenging malignancy due to its pronounced inter- and intra-patient heterogeneity, which contributes to therapeutic resistance and poor clinical outcomes. Capturing and modeling this complexity is essential for the development of effective, personalized therapeutic strategies.

**Methods:**

To investigate molecularly and functionally BLC heterogeneity, this study employed three-dimensional patient-derived organoids (PDOs) and ex vivo tissue slice culture as advanced preclinical models. PDOs were established from a patient’s cohort enrolled at Regina Elena National Cancer Institute in Rome using spatially distinct tumor samples from central (TC) and peripheral (TP) tumor regions to preserve intratumoral heterogeneity. Genomic and transcriptomic fidelity between PDOs and their parental tumors was assessed through multi-omics analyses. Functional assays were conducted to evaluate therapeutic responses. A second patient cohort from the University of Bern, was used to further characterize cellular and microenvironmental features of BLC samples combining ex vivo tissue culture and multiparametric-Flow Cytometry (FACS) to address treatment-induced cancer cell plasticity and epithelial-marker expression dynamics.

**Results:**

PDOs recapitulated the genomic and transcriptomic landscapes of the original tumors. Early passage PDOs retained components of the tumor microenvironment, including immune cell subsets, suggesting their relevance for ex vivo modeling of tumor-immune interactions. Functional assays revealed spatial heterogeneous responses to both chemotherapy and EGFR/FGFR-targeted therapies. A corresponding reduction of the EGFR-high basal-like population was observed in ex vivo tissue cultures. In contrast, treatment with a PD-1 immune checkpoint inhibitor showed consistent responses across PDOs regions but correlated with the degree of immune infiltration observed in the parental tumors.

**Conclusion:**

This integrated dual-cohort approach demonstrates that both BLC PDOs and ex vivo tissue cultures offer a versatile and faithful platform for dissecting BLC heterogeneity and advancing functional precision medicine recapitulating patient-specific immune-tumor interactions observed in native tissues.

**Supplementary Information:**

The online version contains supplementary material available at 10.1186/s13046-026-03701-x.

## Background

Bladder cancer (BLC) is one of the most common cancers worldwide, accounting for approximately 3% of all cancer diagnoses. It is four times more common in men than in women, and it predominantly occurs in individuals over the age of 60 [1]. It is characterized by a high recurrence rate and variable treatment responses, making it a significant clinical challenge [2]. BLC can be classified into non-muscle invasive bladder cancer (NMIBC) and muscle invasive bladder cancer (MIBC). NMIBCs are generally indolent but with a high risk of recurrence, whereas MIBCs tend to metastasize and are associated with high mortality rates [3]. Standard treatments, including transurethral resection of bladder tumor (TURBT), radical cystectomy (RC), and chemotherapy, often result in unpredictable outcomes, even among patients with similar clinicopathological features [2, 4]. This variability highlights the need for more effective treatment approaches that account for the tumor’s complexity to improve survival [2]. In line with this requirement, immunotherapy has recently gained increasing importance in the treatment of advanced or metastatic urothelial carcinoma, with growing evidence of its effectiveness in first-line regimens, leading to better clinical outcomes compared to chemotherapy alone [5].

To further advance treatment efficacy, developing preclinical models that predict clinical responses by elucidating resistance mechanisms and enabling personalized therapeutic strategie has become a central focus in the field of urological research. One of the major advances in this area is the establishment of three-dimensional patient-derived organoid (PDO) models [6]. These self-organizing in vitro systems faithfully recapitulate the three-dimensionality, complexity and functionality of human tumors better than traditional two-dimensional cell lines. PDOs have gained considerable attention as versatile tools in personalized medicine, enabling detailed studies of tumor biology and offering effective platforms for preclinical drug screening [6]. This approach is increasingly being applied to BLC, where PDOs are emerging as valuable models to explore tumor heterogeneity and support the development of patient-specific therapeutic strategies [6, 7]. This study adopts a multicentric approach involving the IRCCS Regina Elena National Cancer Institute (IRE) and the University of Bern (UniBe) to investigate the cellular, molecular and immunological features of BLC PDOs and ex vivo tissue culture. By integrating data from two independent patient cohorts, this study demonstrated the utility of organoids as reliable preclinical platform for evaluating both chemotherapeutic and immunotherapeutic strategies, enabling effective drug priorization for clinical translation. The Bern cohort enabled BLC tissue comprehensive phenotyping, together with ex vivo pharmacological testing on tissue slices. The IRE cohort, in turn, focused on organoid generation and characterization, with therapeutic interventions guided by mutational and immune profiling. More importantly, PDOs were established from both central (TC) and peripheral (TP) regions of orginal tumor to capture intra-tumoral heterogeneity. Together, these complementary approaches demonstrate that early-passage BLC PDOs and ex vivo cultures effectively recapitulate spatial tumor heterogeneity, preserve key immune components, and enable pharmacological modulation of tumor phenotypes, underscoring their role in advancing precision medicine and unraveling BLC complexity.

## Methods

### Patient sample collection and tissue processing

Biological material was obtained from patients diagnosed with bladder cancer undergoing diagnostic TURBT or RC at two institutions: the Department of Urology of the IRCCS Regina Elena National Cancer Institute (IRE, Rome, Italy) and the Inselspital, University Hospital in Bern (Switzerland). All patients provided written informed consent in accordance with current legislation and ethical guidelines, and both studies were approved by the respective Institutional Ethical Committees: Ethic Committee of Rome (approval no. IFO 1767/22); Cantonal Ethical approval Bern (KEK 06/03 and 2017–02295).

For both cohorts, tumor tissues were collected during surgery and processed under sterile conditions to preserve tissue integrity and viability. At IRE, samples included both TP and TC regions obtained during TURBT or RC procedures. Fresh tissue samples (≥ 3 cm^3^) and 10 mL of peripheral blood were collected. Immediately after surgical removal, tumor tissues were placed in sterile containers filled with MACS Tissue Storage Solution (Miltenyi Biotec, 130–100-008) supplemented with 100 U/mL penicillin, 100 μg/mL streptomycin, and 100 μg/mL antimycotic, and maintained at 4 °C for a maximum of 24 h. Tissues were washed in phosphate-buffered saline (PBS) to remove blood and debris. A portion of each sample was stored in RNA later (Thermo Fisher, Carlsbad, CA, USA) at −80 °C for molecular analyses, while the remaining material was minced into small fragments and cryopreserved in MACS Freezing Solution (Miltenyi Biotec, 130–129–552). After 24 h at −80 °C, samples were transferred to liquid nitrogen for long-term storage.

At the University Hospital of Bern, tumor tissues from TURBT or cystectomy were collected in Dulbecco’s MEM medium (Gibco, 61,965–026) supplemented with 100 μg/mL Primocin (InVivoGen, ant-pm-1). For TURBT samples, non-cauterized tissue was selected using cold cup biopsies, whereas during cystectomy, tumor material was collected immediately after bladder removal to prevent hypoxic tissue damage. Tissue resections were divided for the different downstream application: cryopreserved in Fetal Bovine Serum (FBS; Sigma, F7524) containing 10% DMSO (Sigma, D2650) for biobanking, fixed in 4% paraformaldehyde (PFA), and directly processed for ex vivo tissue slice culture.

### Blood processing method

10 mL of whole blood was collected from each patient in BD Vacutainer K_2_EDTA tubes: 1 mL was cryopreserved at −80°, 9 mL was immediately processed to isolate peripheral blood mononuclear cells (PBMCs). The blood sample was diluted to a 1:1 volume ratio with PBS.

Ficoll (CL5020, Lympholyte, Euroclone) was added to a new tube in the same volume as the blood. The diluted blood was gently layered on top of the density gradient centrifugation medium, avoiding mixing the two layers. The tube was centrifugated at room temperature at 800 × *g* for 20 min with the brake off, to separate the different layers (plasma on the top, PBMCs in the interface, Ficoll at the bottom). The PBMC interface was carefully collected by pipetting and washed with PBS by centrifugation at 1200 × *g* for 10 min. The resultant PBMC pellets were resuspended in 3 ml of ammonium-chloride-potassium lysing buffer (07850, STEMCELL Technologies) and incubated on ice for 5 min with gentle mixing to lyse any contaminating red blood cells. The mononuclear cells were washed again with PBS, centrifugated at 1200 × *g* for 10 min. The PBMCs pellet was stored at −80 °C.

### Establishment of PDOs culture

BLC tissues were preserved in MACS Tissue Storage Solution (130–100-008, Miltenyi Biotec) supplemented with 100 U/ml penicillin, 100 μg/ml streptomycin and 100 μg/ml antimycotic for up to 24 h at 4 °C. Afterward, the samples were washed twice in PBS, mechanically minced in a petri dish, and the small fragments were transferred to a T75 flask. Single-cell suspension was obtained using the Tumor Dissociation Kit (130–095–929, Miltenyi Biotec), according to the manufacturer’s protocol. Tissue digestion was performed at 37 °C for 1–2 h pipetting every 15 min, until the larger tissue pieces were broken down. The resulting cell suspension was passed through a 70 µm strainer (Miltenyi Biotec), centrifuged at 300 × *g* for 5 min, washed once in PBS, and centrifuged again at 1200 × *g* for 5 min. If the pellet appeared visibly red, erythrolysis was carried out using Ammonium Chloride Solution (07850, STEMCELL Thecnologies) prior to the wash step. Bladder cancer cells were cultured under two different conditions:

1. Geltrex Matrix (A1413302, Aurogene): the cell pellet was resuspended in cold Geltrex and seeded into a prewarmed 24-well plate at a density of 6 × 10^5^ cells per 30 µl drops. The drops were solidified in a 37 °C, 5% CO2 incubator for 30 min, followed by the addition of 500 µl of organoid culture medium Advanced DMEM/F-12 (12,634,010, Gibco), 200 mM GlutaMAX (35,050,038, Gibco), 1 mM HEPES (15,630,080, Gibco) and growth factors to each well. The medium was refreshed every 2–3 days.
2. Ultra-low attachment (ULA) plates: the cell pellet was directly resuspended in BLC medium supplemented with growth factors and seeded into 24-well ULA plates (662,970, Greiner) at a density of 150,000–250,000 cells per well in 500 µL of medium. Fresh medium was added every 3 days and changed once weekly.

Organoids were passaged depending on the rate of proliferation. PDOs were dissociated into single cells with 5 ml TrypLE (12,605,028, ThermoFisher) at 37 °C for 10–15 min, mixing every 5 min. The cells were then centrifuged at 300 g for 10 min, resuspended in fresh medium, and seeded into a new 24-well plate.

Three different growing media formulations were used for organoid culture:

1. Growth Medium 1: FGF 10, FGF 7, FGF 2, A83-01, Y-27632, B27, N-Acetylcysteine, Nicotinamide (*Mullenders J, *et al*.)*
2. Growth Medium 2: FGF 10, R-Spondin, SB202190, Noggin, Wnt3a, HGF, EGF, A83-01, Y-27632, B27, N-Acetylcysteine, Nicotinamide (*Minoli M, *et al*.*)
3. Growth Medium 3: FGF 10, FGF 7, FGF 2, R-Spondin 1, SB202190, Noggin, Wnt3a, HGF, EGF, A83-01, Y-27632, B27, N-Acetylcysteine, Nicotinamide.

The specific concentrations of the growth factors in the organoid culture media were as follows:

- 10 ng/mL FGF10 (100–26 Peprotech)
- 25 ng/mL FGF7 (HZ-1100, DBA)
- 12.5 ng/mL FGF2 (HZ-1285, DBA)
- 500 ng/mL R-Spondin 1 (HZ-1328-1000UG, DBA)
- SB202190 (S7067, Sigma Aldrich)
- 100 ng/mL Noggin (HZ-1118-1000UG, DBA)
- 10 ng/mL Wnt3a (HZ-1296, DBA)
- 50 ng/mL HGF (HA-1084-100UG, DBA)
- 50 ng/mL EGF (HZ-1326-1000UG, DBA)
- 500 nM A83-01 (S7692-25MG, DBA)
- 1X B27 supplement (17,504,001, Invitrogen)
- 1.25 mM N-Acetylcysteine (A9165, Sigma Aldrich)
- 5 mM Nicotinamide (N0636, Sigma Aldrich)
- 10 µM Y-27632 Rock inhibitor (HY-10583-10MG, DBA).

### Immunohistochemical analysis

Organoid samples were centrifugated at 500 × g for 5 min at 4 °C. Supernatant was discarded and cell pellet was fixed with Preserv-Cyt TM fluid for 20 min before the sample vial with was put in the automated Cellient TM processor suspended with 10 ml of methanol-based Thinprep™ solution. Afterwards, samples were processed by The Cellient™ Automated Cell Block System (Hologic Corporation, Marlborough, MA) which is fully automated and creates a paraffin-embedded cell block using isopropanol for dehydratation and xylene for clarification.

Biopsies of human bladder urothelial carcinoma were fixed in 10% formalin for 24 h and routinely processed into paraffin blocks, formalin fixed-paraffin embedded (FFPE).

Tissue and cell blocks were cut at 3 μm using a microtome LEICA SM 2000R (Advanced Research Systems Inc., Macungie, PA) and mounted onto slides. One section was dewaxed in xylene and rehydrated through a series of graded ethanol solutions and stained with Gill’s Hematolylin (Bio-optica, Milan) and Eosin (Bio-optica, Milan) for microscopic evaluation of specimen cellularity.

Immunohistochemestry (IHC) was performed on the remaining unstained slides which were incubated with the primary antibody in an automated immunostainer (Bond-III, Leica, Biosystems, Italy). A citrate buffer, pH 6 or pH 8, was used to unmask the antigens in each case. The primary antibodies used are listed in Supplementary Table 1.

Images were obtained at × 40 magnification by using Aperio Image Scope system equipped with a Digital Image Capture software. The evaluation was based on the percentage of positive cells.

### Whole Exome DNA Sequencing (WES)

Genomic DNA was quantified using Qubit dsDNA BR Assay Kit (Invitrogen, Carlsbad, CA, USA). Quality was determined (DIN range from 1 to 10) on 4200 TapeStation using Genomic DNA screenTape assay (Agilent Technologies, Santa Clara, USA).

Pre-enrichment libraries were performed using 100 ng of DNA according to Library Preparation EF 2.0 with Enzymatic Fragmentation and the Twist Universal Adapter System (Twist Bioscience, San Francisco, CA, USA) according to the manufacturer’s instructions. Exome hybridization was conducted using a Twist Comprehensive Exome kit (Twist Bioscience, CA) according to the manufacturer’s protocol. This protocol provides coverage for more than 99% of protein-coding genes. The quality of the libraries was assessed using the Agilent 4200 TapeStation system (High Sensitivity D1000 ScreenTape assay), while their quantity was measured by qPCR. The exome library was sequenced on the Illumina NovaSeq 6000 (Illumina, San Diego, CA, USA) platform with 100 bp paired-end reads.

Variant calling was performed setting a cut-off for the variant allele frequency (VAF) at 5%.

### RNA sequencing (RNA-seq)

RNA was extracted from fresh frozen tissues and organoids using AllPrep DNA/RNA/miRNA Universal Kit (80,224, QIAGEN) according to the manufacturer’s instructions. The quality of the RNA has been controlled on a Bioanalyzer with the RNA 6000 Nano kit (Agilent Technologies, Santa Clara, CA, USA). The libraries for the RNA-Sequencing have been prepared using the Illumina Stranded Total RNA Prepwith Ribo-Zero Plus kit following the manufacturer’s instructions. The resulting libraries have been quality controlled on a Bioanalyzer with the High Sensitivity DNA Kit (Agilent Technologies, Santa Clara, CA, USA) and quantified by qPCR. The sequencing has been performed on a NovaSeq 6000 instrument (Illumina Inc., San Diego, CA, USA), sequencing in paired-end mode (2 × 100 bp).

### Digital PCR

Custom-designed primers and probes were generated according to the specific mutation profiles of the cells, with synthesis performed by Integrated DNA Technologies (IDT). Each probe was synthesized as an Affinity Plus® Probe and purified by HPLC to ensure optimal quality.

The samples were analyzed in the same experiment using the Quant Studio™ Absolute Q Digital PCR chip/plate-based system (Life Technologies). Reactions were prepared in a final volume of 10 μL, including 2 μL of 5 × Master Mix, 0.5 μL of 20 × TaqMan® probe, 7.5 μL of template and loaded onto dPCR chips. The thermal cycles were as follows: 10 min at 96.0 °C, 39 cycles at 96 °C for 5 s and 15 s at 60 °C. FAM and VIC fluorescence threshold values were calculated automatically by ThermoFisher Software, reviewed manually, and then applied to the corresponding DNA.

### Organoid treatment and viability assay

At passage 1, organoids were dissociated into single cells with TrypLE and seeded for treatments. 20,000–40,000 cells were seeded in 100 µl of BLC medium into 96-well ULA (650,970, Greiner). Allowed organoids growth, they were treated for 72 h with following compounds from Selleck Chemicals: Cisplatin (1–5–10 µM) (S1166), Gemcitabine (7–14–140 µM) (S1714), Cisplatin + Gemcitabine (1–5–10 µM CDDP + 7–14–140 µM GEM), Erdafitinib (0.01–0.1–1–10 µM) (S8401), Nivolumab (10–20–40 μg/mL) (A2002). For the treatment, 50 µl of BLC medium with the drug dose are added to each well. After 72 h of drug treatment, organoids viability was assessed using ATPlite assay (Revvity, Massachusetts, USA), according to the manufacturer’s instructions. The luminescence was measured using the EnSpire Multimode Plate Reader (PerkinElmer).

### Bioinformatic analysis

WES data were processed with Sarek version 3.4.0, an nf-core pipeline [8] designed for the identification of germline and somatic variants. Gene and variant annotations were performed using the Variant Effect Predictor (VEP), while oncogenic and clinically relevant mutations were identified with the Personal Cancer Genome Reporter (PCGR) tool. Mutation sites were selected based on specific criteria: a minimum of 20 sequencing reads and a minor allele frequency (MAF) of 0.5 or greater. CNVkit v0.3.5 was employed to analyze sequencing coverage and copy number variations, while CNAqc v1.0.0 [9] was used to visualize the loss of heterozygosity (LOH) status.

RNA-seq data were analyzed using the “rnaseq” version 3.9 pipeline integrated into the nf-core platform [10] with default parameters [11]. The Pearson's correlation test was executed in R to assess the relationship between tissue samples and the corresponding patient-derived organoids (PDOs). Unsupervised hierarchical clustering of the pathways was performed by calculating a score with the ssGSEA function in R. The ComplexHeatmap R package was used for both unsupervised hierarchical clustering and Oncoprint visualization. Cell-type fractions were estimated using the Cibersort tool with default parameters; the bpxplot was generated with “ggplot2” R package and the significance was assessed by Wilcoxon test.

### Ex vivoculture

Fresh BLC tissue collected in Basis Media was washed twice and cut into slices of approximately 1 mm of thickness. To preserve as much fidelity as possible to the parental tissue, at least 3 slices per condition were cultured. Treatment groups were defined according to the initial amount of the biological material. Tissue slices were carefully placed on a transwell plate onto 0.4 μm nitrocellulose membranes (ThinCert Greiner Bio-one, 662,640) positioned in a six-well plate filled with 1.5–2 mL of culture medium (DMEM, high glucose, GlutaMAX™ (ThermoFisher, 10,569,010), with 10% FBS (Sigma, F7524), 1% penicillin–streptomycin, 100 μg/mL Primocin (InVivoGen, ant-pm-1), 2.5 μg/mL fungizone (InVivoGen, ant-fn-2). While the tissue is continuously in touch with the liquid but not submerged, nutrient exchange occurs by diffusion from the medium across the membrane. Treatments and vehicles were directly added to the culture medium. Plates were inserted in a sealed container system flushed for 3 min with O_2_ (3 L/min) and incubated at 37 °C for 3 days with the exception of BLC180 for which the treatment was extended to 5 days. At the end of the experiment, tissue slices were collected and cryopreserved for downstream flow cytometry analysis. When available, representative tissue slices were fixed in 4% paraformalaldheyde and embed in paraffin blocks. Briefly tissue slices were processed with HistoCore PEARL-Tissue processor (Leica-Biosystems) and embed in paraffin blocks with the HistoCore ARCADIA- embedding station (Leica Biosystems). 4 μm thickness sections were cut and used for Hematoxylin and Eosin (H&E) and immunohistochemistry (IHC) staining. After deparaffinization and rehydration with Xylol followed by serial dilutions of Ethanol, sections were stained with H&E for general assement of tissue architectural structure. Concerning Ki67 IHC after deparaffinization and rehydratation, antigen retrieval was performed in Citrate-based buffer solution in a high-pressure water bath for 10 min. Non-specific antigen binding blocking was done using 1% bovine serum albumin (BSA)/PBS1x-Tween20 (0.1%) for 1 h at room temperature. The primary antibody, Ki-67 (Cat. No. GTX16667, GeneTex, US), was incubated at a 1:400 dilution in blocking buffer, overnight at 4ºC. Tiusse section were further incubated with anti Rabbit EnVision + System HRP (K4003, DAKO Agilent, US). Lastly, AEC chromogen/substrate (#K050 Diagnostic BioSystems Inc., US) was used and the slides were counterstained with hematoxylin and mounted in EUKITT® mounting medium (HUBERLAB, Switzerland).

### Flow cytometry analysis

Single-cell suspensions derived from tumor tissues and PDOs were analyzed by flow cytometry to characterize both tumor and immune cell populations in the IRE and Bern cohorts. At IRE, tissues and PDOs were dissociated into single-cell suspensions by combining mechanical and enzymatic digestion, as previously described, using the Miltenyi Tumor Dissociation Kit. Cells were washed and stained with LIVE/DEAD Fixable Violet Dead Cell stain (Thermo Fisher Scientific) to assess viability. To prevent nonspecific antibody binding, Fc receptors (CD16/CD32) were blocked using TruStain FcX Plus (BioLegend, CA, USA). Tumor and immune cells were detected using the following antibodies: CD45/BV510 (BHI30) from BD Biosciences (NJ, USA); EpCAM/AF700 (9C4), CD3/FITC (UCHT1), PD-1/BV605 (EH12.2H7), and the corresponding isotype control IgG BV605 from BioLegend (CA, USA). Stained cells were acquired using the Beckman CytoFLEX S flow cytometer (Beckman Coulter, CA, USA), and data were analyzed with FlowJo software v10.10 (BD Biosciences). For data processing, the cell population was first identified using SSC-A/FSC-A dot plots. Doublets were excluded by FSC-A/FSC-H and SSC-A/SSC-H gating, and viable cells were selected by excluding LIVE/DEAD⁺ events. Among live cells, EpCAM⁺ and CD45⁺ populations were identified as tumor and immune cells, respectively. Within CD45⁺ cells, CD3⁺ T cells were further analyzed for PD-1 expression to assess immune checkpoint activation. At the University of Bern, tissue fragments of parental tissue or ex vivo cultured slices were digested with 5 mg/mL Collagenase type II (Gibco, Thermofisher) supplemented with 15 μg/mL DNase I (Roche) and 10 μM Y-27632 (Selleckchem) for 1–2 h at 37 °C with gentle mixing every 20 min. Following digestion, the cell suspension was washed (400 g, 5 min), treated with erythrocyte lysis buffer (NH_4_Cl 150 mM, KHCO_3_ 10 mM, EDTA 0.1 mM) for 10 min at room temperature, and then incubated with TrypLE (ThermoFisher, 12,605,028) for 10 min at 37 °C. The resulting single-cell suspension was filtered through a 50 μm strainer (CellTrics, 040042327), washed, and counted prior to further use.

Tumor-derived single cells were stained with single or multiplexed antibody panels. Cells were resuspended in FACS buffer (0.5% BSA, 2 mM EDTA in PBS, pH 7.4) containing the antibody mix and incubated for 30 min at room temperature in the dark. After incubation, cells were washed and resuspended in 500 μL of FACS buffer supplemented with 5 μg/mL DAPI and kept on ice in the dark until acquisition using a BD FACS SORP LSR II flow cytometer. Flow cytometry data were analyzed using FlowJo v10.10 (Tree STAR, Ashland, OR, USA). Data analysis was performed through a sequential gating strategy. For quality control, FlowAI plugin was used after data acquisition to identify and exclude potential anomalous events caused by irregular flow rate, signal instability, or dynamic‑range issues. To minimize spillover, fluorescence minus one (FMO) controls was performed for the primary gating markers, CD45 (immune), EPCAM (epithelial), and CD31 (endothelial), along with antibody capture compensation beads (AbC™ Total Antibody Compensation Bead Kit, ThermoFisher Cat#A10497) to calculate the spillover spreading matrix. Subsequently, cells were identified using SSC-A/FSC-A plots followed by doublets exclusion via FSC-A/FSC-H gating, and cell death exclusion in DAPI-A/FSC-A plots by excluding DAPI⁺ events. After identifying viable cells, the metadata from all ex vivo samples were concatenated into a single fcs file to allow for joint analysis and comparability between in vitro treatments. A sequential hierarchical gating strategy was used to define the main cellular compartments. CD45 + were defined as immune cells. Then, within CD45- population, EPCAM + defined epithelial cells, while EPCAM- were further subdivided into endothelial (CD31 +) and stromal fractions (CD31-). Dimensionality reduction was performed using the t-distributed stochastic neighbor embedding (tSNE, FitSNE) algorithm. Afterwards, FlowSOM clustering was performed on the CD45-EPCAM + concatenated data using a panel markers to define the epithelial identity (UPKII, FGFR3, CD90, BCAM, CD49f, Claudin-4, Nectin-4, PCAD, CD44 and EGFR). The list of antibodies used in both cohorts is reported in Supplementary Table 2. FlowSOM clustering was summarized in a spanning tree diagram, along with a heatmap illustrating population’s median intensity. Subsequent analyses were performed on the concatenated dataset, with sample‑wise stratification to facilitate visualization and comparisons.

## Results

### A dual-cohort approach: workflow and bladder cancer patient features

**Fig. 1:**
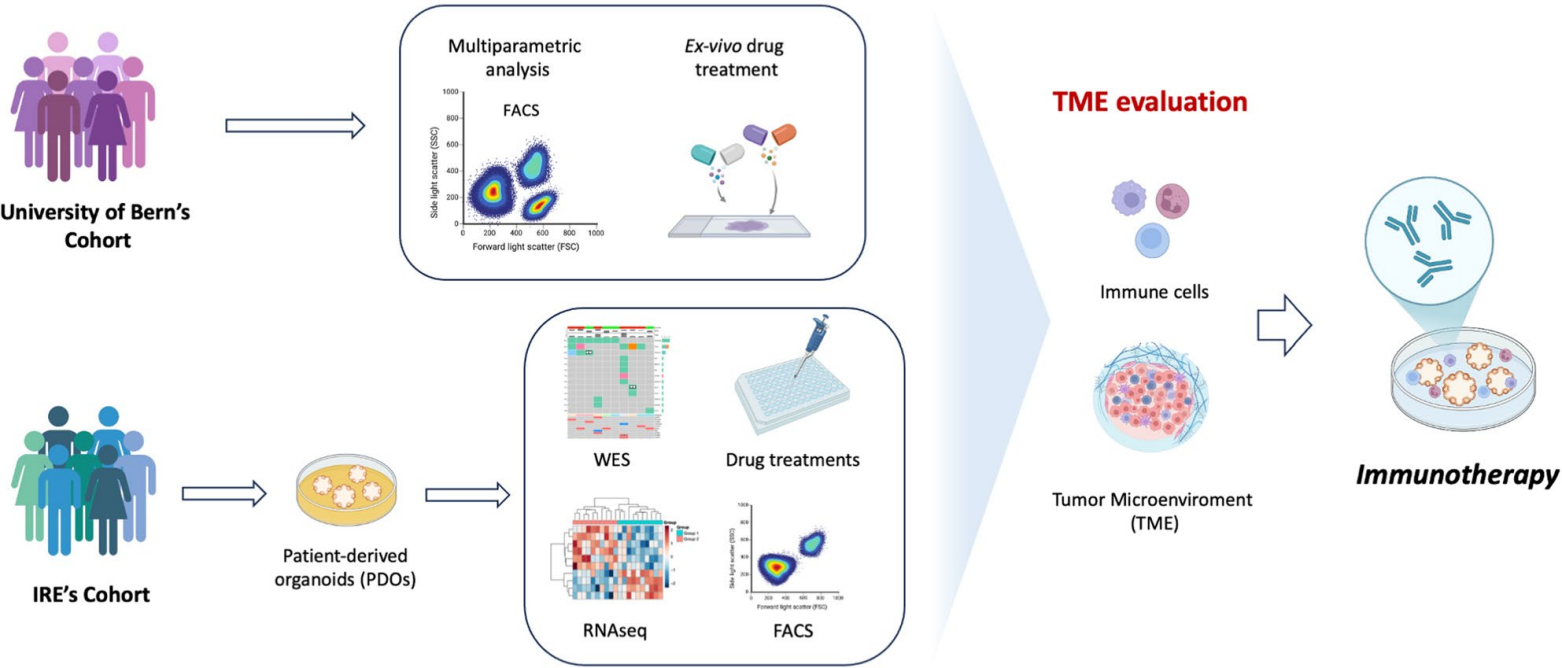
Schematic representation of the workflow

This study is based on a multicentric approach analysing two BLC patient cohorts: one from the IRCCS Regina Elena National Cancer Institute (IRE) in Rome, Italy, and the other from University of Bern (UniBE) in Switzerland. By integrating data from different geographic populations, this approach provides a more comprehensive insight into BLC. The clinical features of both cohorts are summarized in Table 1 (IRE) and Table 2 (UniBE). Consistent with existing literature, urothelial carcinoma is the predominant histopathological subtype in both cohorts. In addition, BLC predominantly affects male patients, with the average age at diagnosis of 70 [2]. A graphical workflow of this study is reported in Fig. 1: the UniBE cohort was used for multiparametric analyses and ex vivo pharmacological treatments on tissue slices focusing on the epithelial compartment, whereas the IRE cohort was primarily employed for the generation and characterization of BLC PDOs, as well as for pharmacological screening. This combined approach revealed the presence of immune and tumor microenvironment (TME) components in BLC tissues and derived 3D models, highlighting their potential as a valuable in vitro tool for immunotherapy and celluar heterogeneity studies.

**Table 1:** Clinical features of IRE’s Cohort (*N* = 15)

Sample ID	Type	Sample type	Tumor stage	Gender	Histological variant
BLC15	MIBC	Cystectomy	T4a N1 HG	Male	Urothelial carcinoma
BLC17	MIBC	Cystectomy	T2a HG	Male	Urothelial carcinoma
BLC32	MIBC	Cystectomy	T2a pN0 HG	Male	Urothelial carcinoma
BLC38	NMIBC	TURBT	Ta LG	Male	Urothelial carcinoma
BLC41	MIBC	Cystectomy	T2b N0 HG	Male	Urothelial carcinoma
BLC50	MIBC	Cystectomy	T4a	Female	Squamouscarcinoma
BLC51	MIBC	Cystectomy	T2a N0 HG	Male	Urothelial carcinoma
BLC63	NMIBC	TURBT	T1 HG	Male	Urothelial carcinoma
BLC78	MIBC	Cystectomy	T3b N0 HG	Female	Urothelial carcinoma
BLC82	MIBC	Cystectomy	T3b HG	Male	Urothelial carcinoma
BLC92	MIBC	Cystectomy	T3b	Female	Squamouscarcinoma
BLC110	MIBC	Cystectomy	T3b N0 HG	Male	Urothelial carcinoma
BLC111	MIBC	Cystectomy	T3b N2	Male	Neuroendocrine carcinoma
BLC112	MIBC	Cystectomy	T2a N0	Female	Urothelial carcinoma
BLC117	MIBC	Cystectomy	T2 HG	Male	Urothelial carcinoma

**Table 2:** Clinical features of University of Bern’s Cohort (*N* = 18)

Sample ID	Type	Sample type	Tumor stage	Gender	Histological variant
BLC179	NMIBC	TURBT	Ta HG	Male	Urothelial carcinoma
BLC146	NMIBC	TURBT	T1 HG	Male	Urothelial carcinoma
BLC180	NMIBC	Nephroureterectomy	T1 HG	Female	Urothelial carcinoma
BLC46	NMIBC	TURBT	≥ T1 HG	Female	Urothelial carcinoma
BLC86	MIBC	Cystectomy	T2b N0 M0	Male	Urothelial carcinoma
BLC174	MIBC	Cystectomy	T2b N0 M0	Male	Urothelial carcinoma
BLC98	MIBC	Cystectomy	T3a N0 M0	Male	Urothelial carcinoma
BLC163	MIBC	Cystectomy	T3a N0 M0	Female	Squamous carcinoma
BLC159	MIBC	Cystectomy	T3b N0 M1	Male	Urothelial carcinoma
BLC176	MIBC	Cystectomy	T3b N1 M0	Male	Urothelial carcinoma
BLC159	MIBC	Cystectomy	T3b N0 M1	NA	Urothelialcarcinoma
BLC175	MIBC	Cystectomy	NA	NA	NA
BLC177	MIBC	Cystectomy	T3b N1 M0	Male	Urothelialcarcinoma
BLC193	MIBC	Cystectomy	T1 HG	Female	Sarcomatoid/urothelialcarcinoma
BLC194	NMIBC	Nephroureterectomy	Ta LG	Female	Urothelial carcinoma
BLC227	MIBC	Cystectomy	NA	NA	NA
BLC228	MIBC	Cystectomy	NA	NA	NA
BLC229	MIBC	Cystectomy	NA	NA	NA

*NA* Info not available, *MIBC* muscle invasive bladder cancer, *NMIBC* non-muscle invasive bladder cancer, *LG* low grade, *HG* high grade

### Establishment and characterization of PDOs from IRE cohort to investigate BLC heterogeneity

A cohort of 118 BLC patients underwent either diagnostic TURBT or RC, were enrolled at IRE, yielding 85 tissue samples (72%) from RC and 33 (28%) from TURBT. Comprehensive samples were collected from each patient, encompassing TC and TP tissues, for the generation of PDOs.

Significant efforts have been made to optimize PDO culture conditions. Single cells cultured in ultra-low attachment (ULA) plates exhibited a higher success rate compared to those in Geltrex (60% vs. 17%; p < 0.001) (Supplementary Fig. 1a-b), with no significant differences observed between fresh and frozen tissues or between peripheral and central tumor regions (Supplementary Fig. 1b). Notably, the use of Growth Medium 3 significantly enhanced PDOs generation (60%; p = 0.02). Although Growth Medium 3 contains selected components from both Growth Medium 1 and 2, experimental observations indicated that its improved performance was primarily associated with the presence of the Wnt-enhancing cytokines R-spondin, Noggin and Wnt3a. Media lacking this cytokine triad (such as Growth Medium 1) consistently showed lower organoid establishment efficiency, suggesting that activation of Wnt signalling combined with inhibition of BMP pathways represents a key determinant for BLC PDO initiation.

Overall, 31 PDOs were successfully generated from 53 tissue samples, reflecting a success rate of 60%.

A subset of 12 representative PDOs, comprising 2 from TURBT, and 10 from RC, was extensively characterized through Whole Exome Sequencing (WES) and RNA sequencing (RNAseq) within 7 days of culture initiation. These analyses demonstrated a strong correlation between PDOs and their matched tissues concerning mutational and transcriptional profiles in most patients (Supplementary Fig. 1c-e).

**Fig. 2:**
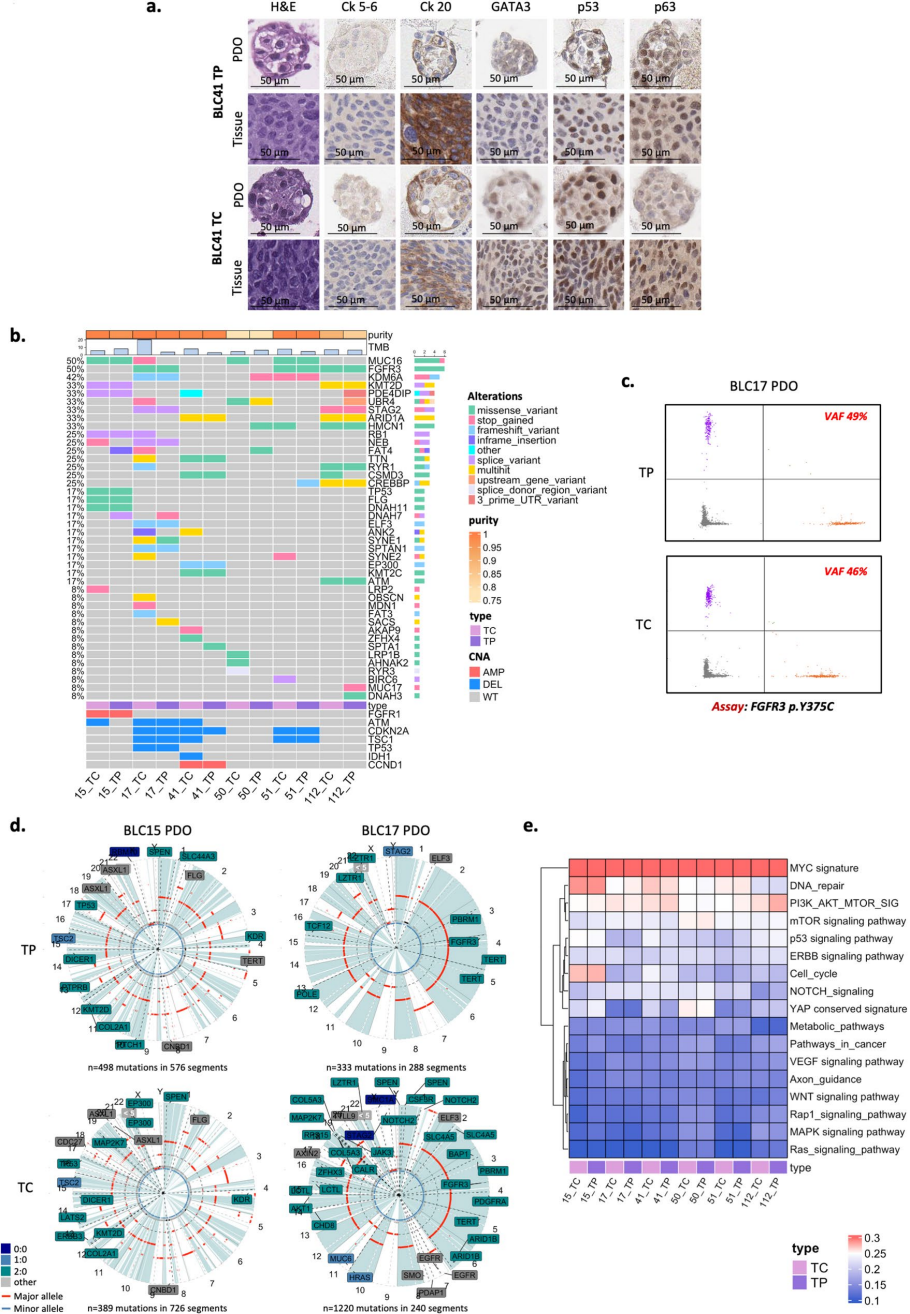
Establishment and characterization of bladder cancer PDOs from central and peripheral tumor tissues. **a** Comparative Haematoxylin and Eosin staining and immunohistochemistry staining for indicated markers of BLC organoids and matched tissues (BLC41). **b** Oncoplot of top mutated genes in BLC from WES analysis in TC and TP PDOs samples. Tumor purity and TMB are reported on the top. **c** 2D dPCR analysis of matched PDOs (BLC17 TP, BLC17 TC) for FGFR3 p.Y375C mutation. Orange, purple, green and grey dots depict wild-type, mutated, double-positives and not amplified dPCR spots, respectively. VAFs are indicated. **d** Comparative LOH status between matched TP and TC organoids (BLC15, BLC17)*. ***e** ssGSEA pathway heatmap of cancer pathways expression in matched TP and TC PDOs. BLC: bladder cancer; PDO: patient-derived organoids; WES: whole exome sequencing; TC: central tumor; TP: peripheral tumor; CK: cytokeratin; LOH: loss of heterozygosis; VAF: Variant allele frequencie; ssGSEA: single sample gene set enrichment analysis

To deeper explore BLC heterogeneity and region-specific tumor behaviours, PDOs were generated from two distinct tumor regions, TP and TC, in six patients (BLC15, BLC17, BLC41, BLC50, BLC51, BLC112) who underwent RC. Morphological characterization of these PDOs and matched tissues was assessed through immunohistochemistry (IHC) staining evaluating the expression of p53, basal markers (cytokeratin (Ck) 5/6 and p63), and luminal markers (Ck20 and GATA3). Representative images from sample BLC41 are shown in Fig. 2a (see also Supplementary Fig. 2 for additional samples). The findings obtained revealed histological similarities between both TP and TC PDOs and their corresponding tissue samples. Notably, TP tissues and their matched PDOs showed lower expression levels of BLC markers compared to the corresponding TC samples.

To explore the mutational status of TP and TC PDOs, the WES analysis was performed. By analyzing The Cancer Genome Atlas (TCGA) dataset (*n* = 411 patients), the top mutated genes in BLC (*n* = 43) were identified, as depicted in the oncoplot with different color-coded genomic alterations (Fig. 2b). WES revealed some differences between TP and TC PDOs samples, including the loss of certain mutations in TP that were present in TC (Fig. 2b). This was particularly evident in the BLC17 sample, where the TP sample exhibited a loss of specific mutations compared to TC, accompanied by a significant reduction in the tumor mutational burden (TMB) (from 20.03 mut/Mb to 3.79 mut/Mb) (Supplementary Table 3). Mutations retained in both TP and TC samples displayed similar variant allele frequencies (VAF) (Supplementary Table 4). For instance, in the BLC17 sample, the FGFR3 p.Y375C mutation, a known druggable target in BLC, exhibited comparable VAFs in both regions (Supplementary Table 4). This similarity was validated using digital PCR (dPCR) (Fig. 2c).

The loss of heterozygosity (LOH) analysis revealed a significant reduction in LOH status within TP samples compared to TC samples (Fig. 2d).

The RNA-seq analysis that focuses on cancer-related pathways, demonstrated a high degree of transcriptional similarity between TC and TP samples (Fig. 2e). In particular, the Single Sample Gene Set Enrichment Analysis (ssGSEA) revealed similar expression levels of key cancer pathways, including MYC signature, PI3K/AKT/MTOR signalling, WNT and p53 pathways (Fig. 2e).

These findings suggest that while TC and TP PDOs share similar transcriptional profiles, they differ at the mutational level, highlighting the unique molecular landscapes of peripheral and central tumor regions. This underscores the importance of region-specific analyses to fully understand the heterogeneity of BLC.

### Evaluation of BLC PDOs response to standard chemotherapy and target therapy

**Fig. 3:**
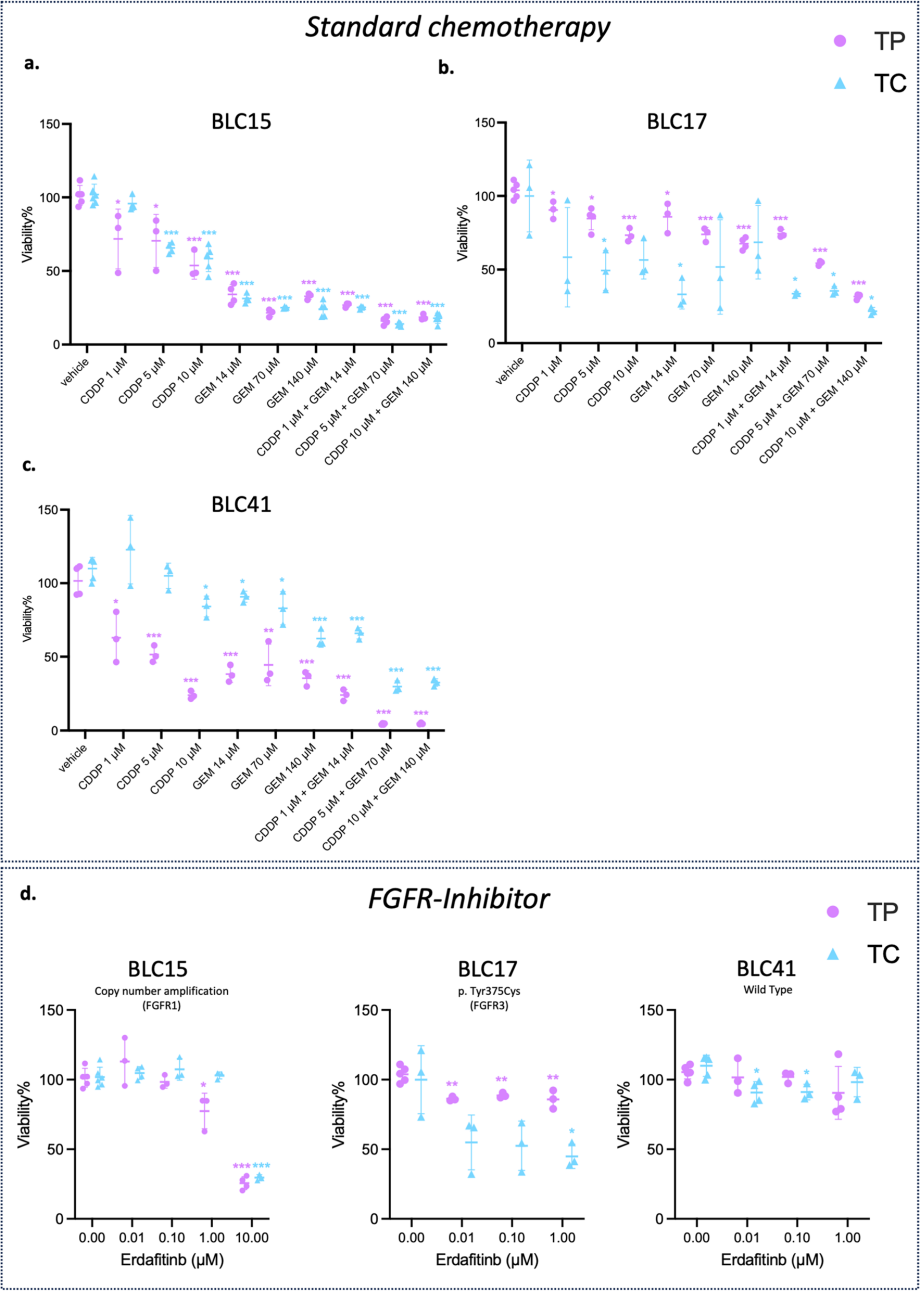
BLC PDOs response to standard chemotherapy and target therapy. **a**-**c** Dot plot of viability assay (%) evaluated by ATPlite analysis of matched TP (purple) and TC (blue) PDOs response to 72 h of treatment with CDDP and GEM at the indicated concentrations (BLC15, BLC17, BLC41). **d** Dot plot of viability assay (%) evaluated by ATPlite analysis of FGFR-mutated PDOs (BLC15, BLC17) response to 72 h of treatment with Erdafitinib (FGFR-inhibitor) at the indicated concentrations, compared to FGFR wild-type PDOs (BLC-41). TC: central tumor; TP: peripheral tumor; BLC: bladder cancer; PDO: patient-derived organoids; CDDP: cisplatin; GEM: gemcitabine

A crucial aim was to determine the feasibility of using BLC PDOs as a tool for testing the response to therapies in vitro. To achieve this, PDOs were treated with standard BLC chemotherapy, specifically cisplatin (CDDP) and gemcitabine (GEM), both individually and in combination, across a range of clinically relevant concentrations (Fig. 3a-c). PDOs viability was assessed after 72 h of treatment. Figure 3a-c presents a comparative analysis of chemotherapy response in matched TP and TC PDOs from three representative samples (BLC15, BLC17, BLC41). The results revealed variable responses between TP and TC PDOs across all different patient samples. In BLC15 samples, both TP and TC PDOs exhibited similar response to chemotherapy (Fig. 3a). Conversely, BLC17 samples demonstrated that TC PDOs had a stronger response compared to TP PDOs (Fig. 3b). In BLC41 samples, the opposite trend was observed, with TP PDOs showing a more robust response than TC PDOs (Fig. 3c).

Subsequently, the efficacy of target therapy was evaluated by treating TP and TC PDOs with the FGFR-inhibitor, erdafitinib. Figure 3d shows the comparative cell viability data of BLC15, BLC17, BLC41 samples. Particularly, BLC17 harbours an FGFR3 missense variant mutation (p.Y375C), while BLC41 lacks FGFR3 alteration, that serves as a control (Supplementary Table 4). Both TP and TC PDOs were treated with increasing doses of erdafitinib for 72 h. The results demonstrated that the observed heterogeneity in response to chemotherapy persisted with target therapy treatment. BLC17 TC PDOs exhibited a 50% reduction in cell viability with increasing drug concentrations, whereas BLC41 PDOs that lack FGFR3 alterations, showed minimal changes in viability, remaining approximately a 100% reduction (Fig. 3d).

Interestingly, despite both TP and TC PDOs from BLC17 carrying the same FGFR3 mutation at similar VAF (Supplementary Table 2), BLC17 TP PDOs showed a poor response to erdafitinib, suggesting that factors beyond the presence of the mutation, such as alterations in cancer signalling pathways or any influence from the TME, may impact drug sensitivity (Fig. 3d). Given that erdafitinib is a pan-FGFR inhibitor, the analysis was extended to BLC15, which features FGFR1 copy number amplification.

Compared to BLC17, BLC15 exhibited a significant response to treatment in both TP and TC PDOs only at higher drug concentrations, resulting in a 70% mortality rate (Fig. 3d).

These findings highlight the substantial inter- and intra-patient variability in responses to both chemotherapy and target therapy, reflecting the molecular heterogeneity that is inherent in BLC. Consequently, these results emphasize the necessity for personalized and region-specific therapeutic strategies to enhance treatment effectiveness in BLC. ​

### Cytological classification of bladder cancer subtypes is associated with distinct BLC features

**Fig. 4:**
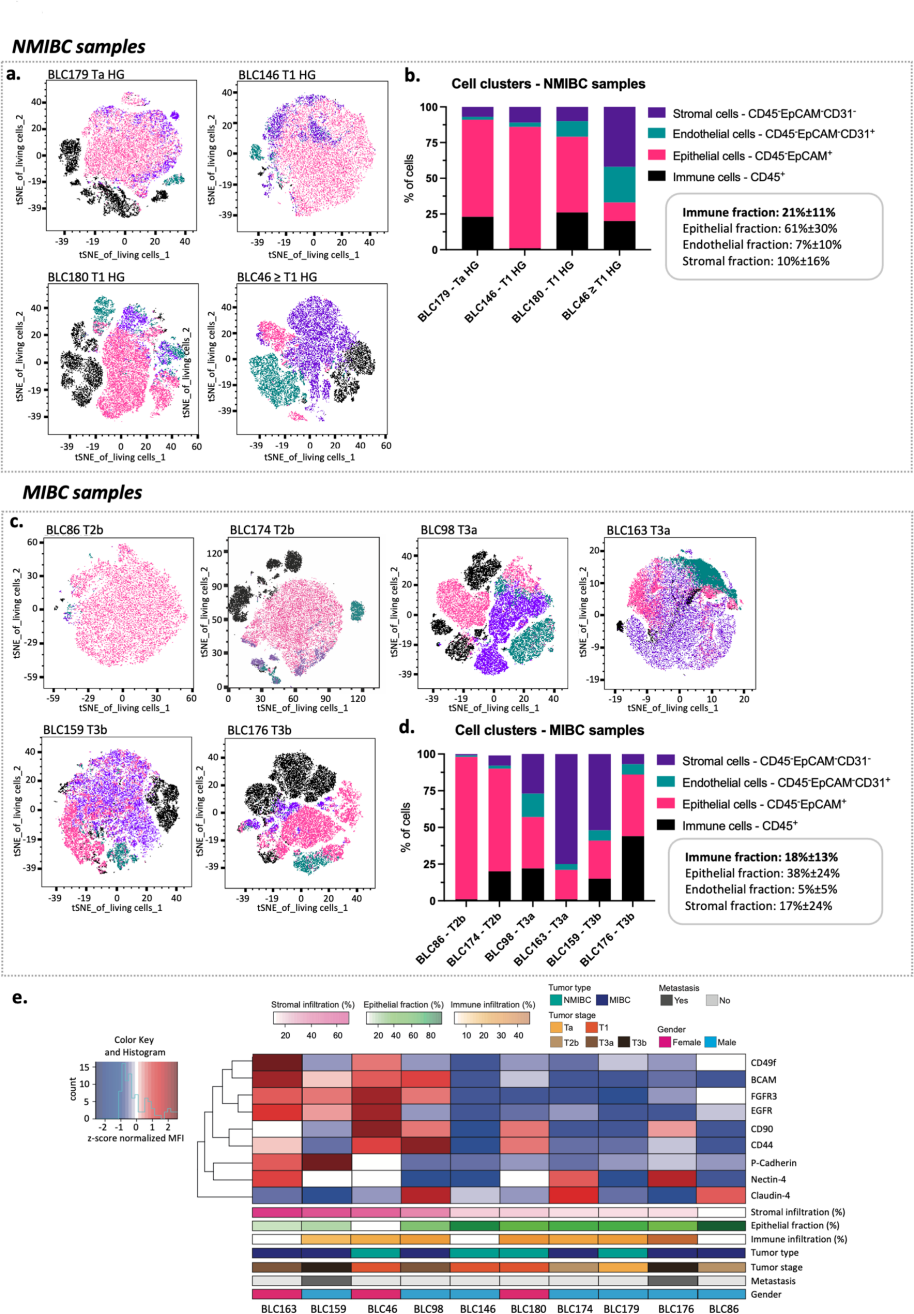
Identification of cell clusters with flow cytometry in NMIBC and MIBC samples. **a**-**d** tSNE representing living cells clustered as stromal, endothelial, epithelial, or immune cells in NMIBC (**a**) and MIBC (**c**) Fraction of living cells from NMIBC (**b**) and MIBC (**d**) samples clustering as stromal, endothelial, epithelial, or immune cells. **e** Heatmap reporting the z-score calculated from normalized mean fluorescent intensity (nMFI) for each marker (MFI * fraction of positive cells). Red indicates overall high expression, whereas blue overall low expression. Samples ordered by stromal infiltration. Unsupervised hierarchical cluster analysis of the markers. Information regarding fraction of stromal infiltration, epithelial fraction, and immune infiltration, as well as tumor type, tumor stage, metastasis and gender are reported in the bottom of the heatmap. NMIBC: non-muscle invasive bladder cancer; MIBC: muscle invasive bladder cancered

Given the marked heterogeneity in drug responses observed in BLC PDOs, the association between these functional differences and intrinsic variations in cellular composition and phenotypic states of native tumors was further investigated by multiparametric FACS. To this end, a representative cohort of 10 treatment-naïve, freshly resected BLC tissues from NMIBC and MIBC patients enrolled at UniBe was analyzed, providing a reference framework for the interpretation of subsequent ex vivo and PDO-based therapeutic screening (Table 2). In particular, a panel of 17 cell surface markers (see Material and Methods) was used to identify four main cell populations —epithelial, immune, stromal, and endothelial— represented in an unsupervised nonlinear dimensionality reduction algorithm (t-SNE) (Fig. 4a and c). The cellular composition varied significantly between samples. As expected, the fraction of epithelial cells was higher in NMIBC samples (61% ± 30%) than in MIBC samples (38% ± 24%) whereas the fraction of stromal cells was slightly higher in MIBC (17% ± 24%) samples than in NMIBC samples (10% ± 16%) (Fig. 4a-d). The immune and endothelial cell fraction was highly variable and not clearly associated with tumor stage. In NMIBC the immune and endothelial fractions were 21% ± 11% and 7% ± 10% respectively, while in MIBC barely dropped to 18% ± 13% and 5% ± 5%, respectively (Fig. 4a-d). Then, the epithelial phenotype panel was compared across all the analyzed samples. To integrate the contribution of the mean fluorescent intensity (MFI) to the positive subset, each epithelial-related marker was plotted as nMFI = (%positive/100)*MFI) for each sample. The heatmap reports the nMFI z-score values across the samples for each marker, revealing high inter-sample heterogeneity (Fig. 4e). Samples exhibiting higher stromal infiltration, exhibited overall higher expression and pronounced heterogeneity of epithelial markers, in particular basal-cell differentiation markers, such as CD49f, BCAM, CD90 and CD44 (Fig. 4e) [12]. Interestingly, this heterogeneity partially reflects the variability observed in the clinical features of this cohort. For example, the histological variant BLC163, a pure squamous carcinoma, expressed the highest levels of basal-differentiated markers, CD49f and BCAM, as expected (Fig. 4e). In addition, the two samples presenting metastasis at diagnosis, expressed the lowest levels of Claudin-4 and were highly immune infiltrated, which is generally associated with lower overall survival (Fig. 4e) [13]. Nevertheless, no similar association was evident across different tumor stages. Taken together, these results suggested that individual molecular variability prevailed over the baseline clinicopathological identity.

### Ex vivo drug testing on BLC tumor slices reveals treatment-specific modulation of cellular composition and marker expression

**Fig. 5:**
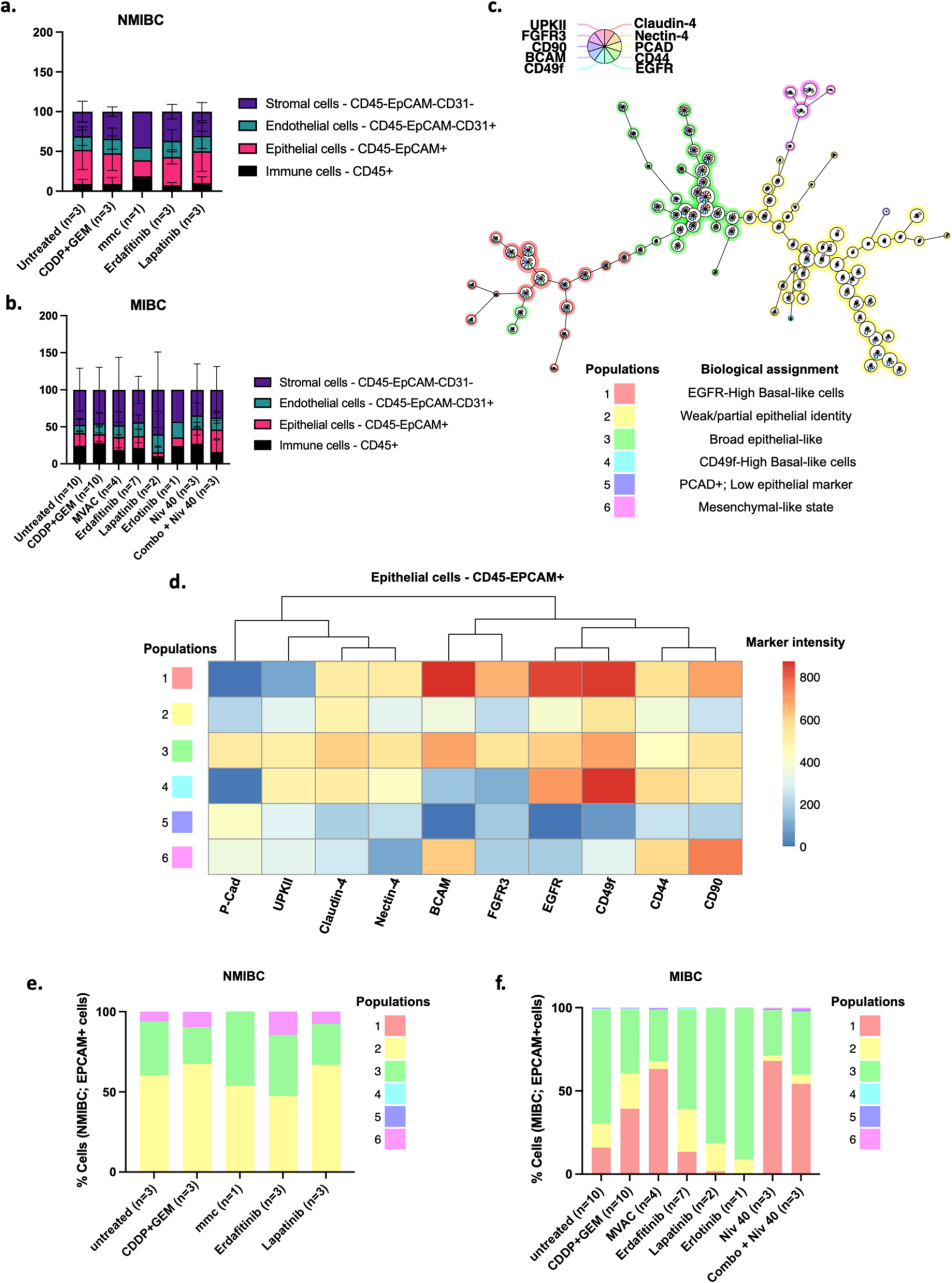
Evaluation of marker expression with flow cytometry on BLC tissue treated ex vivo. **a**-**f** Fraction of living cells from NMIBC (**a**) and MIBC (**b**) ex vivo cultured samples clustering as stromal, endothelial, epithelial, or immune cells. **c** Distinct cellular epithelial populations organized in a spanning tree. **d** The heatmap displays median epithelial markers expression patterns across the concatenated metadata of the ex vivo samples. Populations were annotated based on marker expression profiles and biological characteristics. Fraction of viable CD45-EPCAM + cells from NMIBC (**e**) and MIBC (**f**) according to the distribution of the generated epithelial clusters. For each treatment condition, the sample size is underlined according to the patient sampled. NMIBC: non-muscle invasive bladder cancer; MIBC: muscle-invasive bladder cancer; MMC: mitomycin C; CDDP: cisplatin; GEM: gemcitabine; MVAC: methotrexate, vinblastine, doxorubicin, and cisplatin

A previously developed ex vivo culture system for BLC tumor slices was implemented and further characterized by evaluating the same 17 cell surface markers’ expression in BLC samples, through FCM and histological characterization (Fig. 5) [14, 15]. A total of 13 samples, including both MIBC and NMIBC were used. Standard chemotherapeutics such as CDDP plus GEM, the combination of methotrexate, vinblastine, doxorubicin and CDDP (MVAC) and mitomycin C were used. In addition, experimental targeting compounds, like tyrosine kinase inhibitors that proved to be effective on BLC in vitro – erdafitinib, lapatinib and erlotinib—were included in the screening [7]. Nivolumab was also tested both as a single agent and in combination with CDDP and GEM, a strategy that is gaining clinical relevance [16].

In this experimental design, the parental tissue of origin and the untreated ex vivo tissue slices were used as control conditions. No significant differences in the composition of the main cellular compartments were found between controls (Supplementary Fig. 3). In both “parental” and “untreated” conditions, the NMIBC group generally maintains a higher epithelial fraction, while the MIBC is proportionally enriched in stroma and immune cellular compartments (Supplementary Fig. 3). Similarly, original tissue histo‑architectural features, as well as, Ki-67 expression patterns were reasonably preserved in ex vivo, acknowledging the expected technical variability between sections and low overall proliferation (Supplementary Fig. 4 and Supplementary Fig. 5).

The same analytical pipeline was subsequently applied to the ex vivo cultures to assess the distribution of the main cell types following each treatment. Overall, cellular composition remained comparable across treatment conditions, in both histological subtypes (Fig. 5a and b). Then, to further dissect epithelial heterogeneity, FlowSOM clustering was performed on the CD45-EPCAM + concatenated dataset (*n* = 13 samples), based on multiple epithelial-related markers (UPKII, FGFR3, CD90, BCAM, CD49f, Claudin-4, Nectin-4, PCAD, CD44 and EGFR). Six distinct cell populations were identified and annotated according to their biological signature (Fig. 5c). The shifts in these epithelial clusters in response to the different treatments were then investigated. MIBC displayed a lower epithelial fraction, however greater epithelial-marker heterogeneity compared to NMIBC. Among the identified epithelial clusters, population #2 and #3 were the most abundant ones across the cohort, in both histological subtypes (Fig. 5c-f). These populations represent the “core” epithelial identity with baseline marker expression but no distinct subtype-defining features (Fig. 5c). Consistently, these populations occupied the central region of the spanning tree, reflecting their identical marker signature and prevalence (Fig. 5c). Notably, population #1 was dominated by basal differentiation markers (BCAM and CD49f) together with a pronounced expression of EGFR and moderate intensity of FGFR3 (Fig. 5d). This “EGFR-high basal-like” phenotype was primarily associated with MIBC samples (Fig. 5f). However, its abundance was markedly reduced after treatment with the FGFR3 inhibitor, erdafitinib or the EGFR inhibitors, lapatinib and erlotinib (Fig. 5f). In contrast, an increased representation of this high-basal-like features was observed following chemotherapy or chemotherapy‑based combination treatments, indicating a relative enrichment of cells with basal‑like features under these conditions, highlighting its potential association with resistant traits (Fig. 5f). NMIBC tissues, however, remained largely unchanged in epithelial composition across all treatment conditions (Fig. 5e). Populations #4 and #5 were scarcely detected across the entire series, occurring rarely in MIBC samples and completely absent in the non-muscle invasive group (Fig. 5e and f). Population #4 was dominated by CD49f, primarily defining basal-like progenitor cells with stemness features [17–19] (Fig. 5d). Conversely, population #5 was negative for all the markers, showing only residual expression of P-CAD, which is often associated with increased invasiveness and malignant behavior [20] (Fig. 5d). Finally, population #6, characterized by dominant CD90 expression, consistent with a mesenchymal-like profile, was present in considerable proportion in NMIBC tissues and, to a lesser extent, in MIBC (Fig. 5e and f). This population was absent after treatment with mitomycin C (Fig. 5e). However, these results should be interpreted with caution, due to the variability on the sample size and since this treatment condition was represented by a single patient sample (Fig. 5e). Taken together, treatment with tyrosine kinase inhibitors appeared to effectively modulate their respective targets and to reduce the epithelial identity heterogeneity characteristic of MIBC.

### BLC PDOs retain key features of the tumor microenvironment

**Fig. 6:**
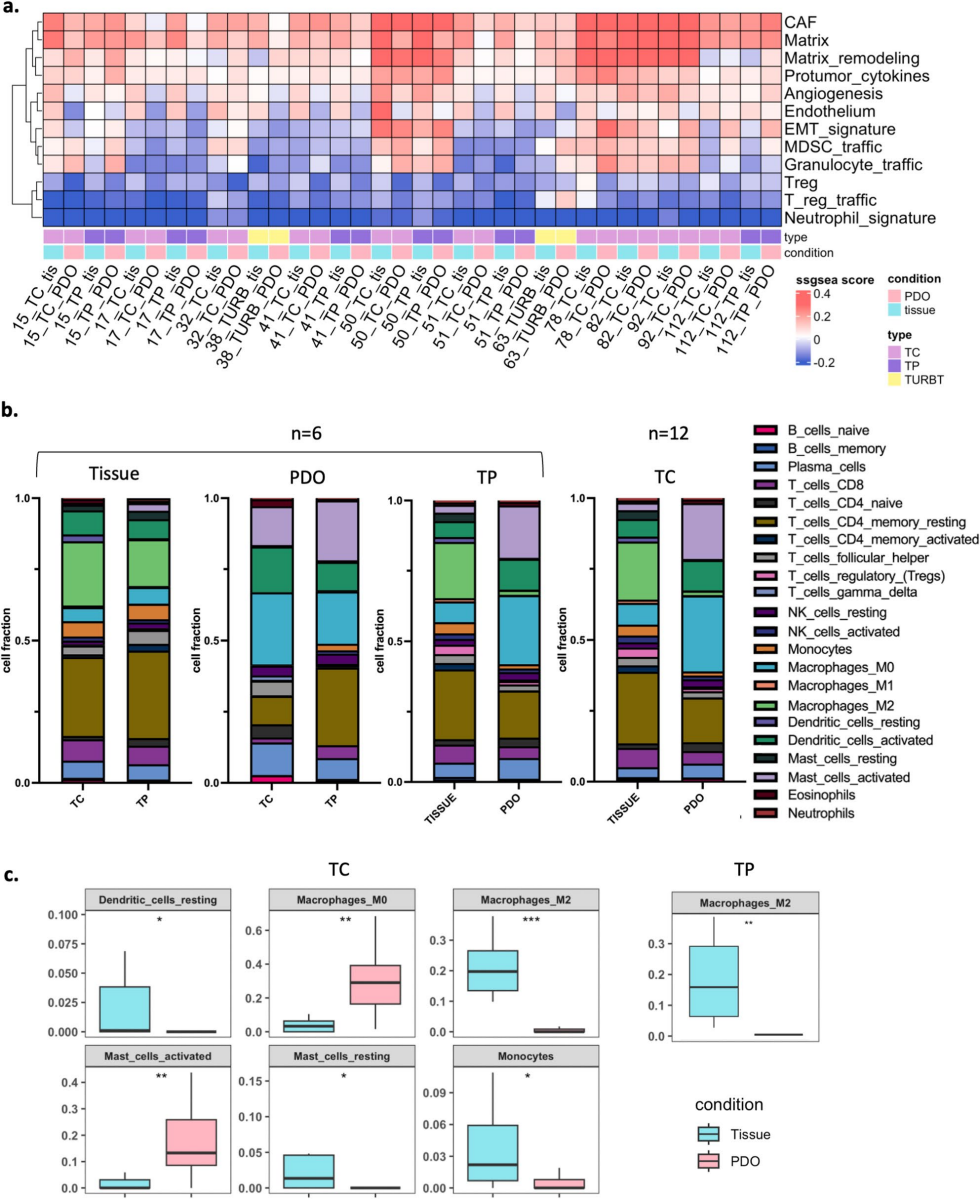
Characterization of BLC tissues and PDOs microenvironment. **a** ssGSEA pathway heatmap of TME pathways expression in matched tissues and PDOs. **b** Deconvolution analysis bar plot of immunity cells fractions performed by Estimate tool*. ***c** Comparison of immune cell populations between PDOs and matched tumor tissues based on deconvolution analysis. Box plots show significantly different immune cell fractions in central (TC, left panel) and peripheral (TP, right panel) samples. TME: tumor microenvironment; TP: peripheral tumor; TC: central tumor; PDO: patient-derived organoids

To further assess the preservation of tumor heterogeneity and microenvironmental features in vitro, BLC PDOs from the IRE cohort were analysed. In particular, a specific ssGSEA was performed on BLC tissues and their matched PDOs (Fig. 6a). The resulting heatmaps provide a comparative overview of TME-related signatures, including extracellular matrix components (Matrix, cancer-associated fibroblasts, Matrix remodelling), vascular processes (Endothelium, Angiogenesis), pro-tumor signalling (Protumor cytokines), and immune components, such as MDSC, granulocyte, and regulatory T cell (Treg) traffic, as well as the neutrophil signature (Fig. 6a).

BLC tissues exhibit a pronounced activation of extracellular matrix-related and vascular pathways, indicative of a highly stromal and angiogenic TME. PDOs largely maintain the overall enrichment pattern of these signatures, however certain differences emerged (Fig. 6a). Notably, extracellular matrix and vascularization pathways remain dominant, but a relative decrease in matrix remodelling and endothelial signatures was observed. Furthermore, alterations in immune-related signatures, particularly in granulocyte and MDSC trafficking, suggest an adaptive response to in vitro conditions (Fig. 6a).

Despite these variations, the neutrophil signature and Treg trafficking keep a consistent suppression pattern across both conditions. These findings indicate that PDOs retain critical transcriptional features of the original tumor tissue while adapting to the in vitro setting, particularly in stromal and immune-related components, which is likely due to the lack of vascularization and systemic immune interactions.

To further characterize the immune landscape of PDOs and their corresponding tissues, a deconvolution analysis was performed, and the relative proportions of various immune cell subsets, including B cells, T cells (CD4 +, CD8 +, regulatory T cells), natural killer (NK) cells, monocytes, macrophages (M0, M1, M2), dendritic cells, mast cells, eosinophils, and neutrophils, were determined (Fig. 6b and Supplementary Table 5).

Comparison of TP and TC tissue samples with their respective PDOs allowed to evaluate immune cell retention in vitro. Tissue samples display a more complex immune composition, with a higher representation of macrophages, dendritic cells, and T cell subsets. Conversely, PDOs show a remodelling of the immune landscape, including a reduction in monocytes and resting NK cells, while certain populations, such as macrophages and CD4 + T cells, remain partially conserved (Fig. 6b). More specifically, the deconvolution analysis revealed some significant differences in immune cell populations between PDOs and matched tumor tissues. PDOs generated from the central tumor area showed increased proportions of macrophages M0 and activated mast cells, while immune components such as macrophages M2, dendritic cells, and monocytes were significantly reduced compared to their matched tissues (Fig. 6c). Similarly, PDOs derived from the peripheral tumor area exhibited a marked reduction in Macrophages_M2 in relation to the corresponding tumor samples (Fig. 6c). Overall, differences in immune cell populations between PDOs and matched tissues were not particularly pronounced, the retention of these immune elements supports the potential of PDOs as reliable surrogates for studying the tumor immune microenvironment as well as for testing immunotherapeutic strategies.

### Early passage BLC PDOs as a tool to evaluate nivolumab-induced immune response

**Fig. 7:**
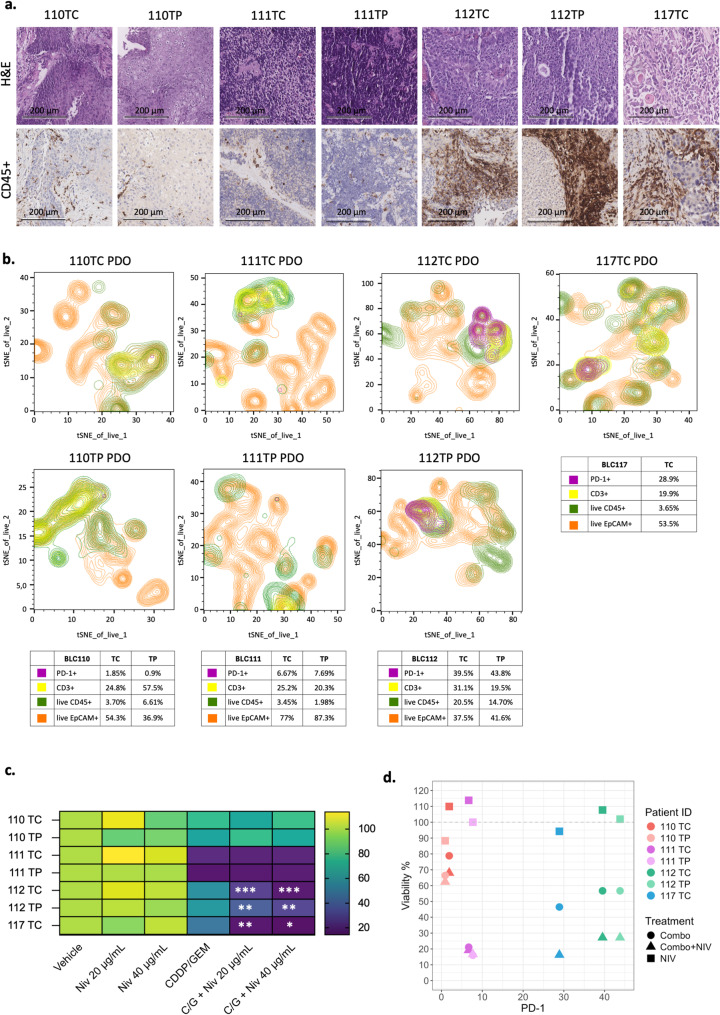
BLC PDOs response to immunotherapy. **a **Comparative immunohistochemistry staining for H&E and CD45 + in BLC110 TP; BLC110 TC, BLC111 TP, BLC111 TC, BLC112 TP, BLC112 TC, BLC117 TC tissues. **b** t-SNE plots showing the distribution of immune (CD45 +, CD3 +, PD1 +) and epithelial (EpCAM +) cell populations in BLC110 TC, BLC111 TP, BLC111 TC, BLC112 TP, BLC112 TC, BLC117 TC PDOs. In the insert below percentage of live EpCAM + CD45 -, EpCAM - CD45 +, CD3 + T lymphocytes (gated in CD45 +) cells and PD-1 + expressing cells (gated in T cells) was reported. **c** Heatmap of viability assay (%) evaluated by ATPlite analysis of BLC110 TC, BLC111 TP, BLC111 TC, BLC112 TP, BLC112 TC, BLC117 TC PDOs response to 72 h of treatment with CDDP, GEM and Nivolumab. Nivolumab 20–40 μg/mL; Combo: CDDP 10 μM + GEM 140 μM. Cell viability is color-coded from high (green) to low (purple). **d** Scatter plot illustrates cell viability following treatment with chemotherapy alone (Combo), chemotherapy combined with Nivolumab at 20 µg/mL (Combo + NIV 20), and Nivolumab alone (NIV 20), relative to the proportion of PD-1 + cells within each sample. Each shape represents a different treatment group (circle: Combo, triangle: Combo + NIV 20, square: NIV 20), and each colour corresponds to a distinct sample. H&E: hematoxylin and eosin; TC: central tumor; CDDP: cisplatin; GEM: gemcitabine; BLC: bladder cancer; PDO: patient-derived organoids

To thoroughly investigate the preservation of the immune component within tissues and matched PDOs, a combined histological, FACS and functional analysis was performed on four samples (Fig. 7). IHC for CD45 + revealed marked variability in immune cell infiltration across tumor tissues: samples 112 TP, 112 TC and 117 TC exhibited a high density of CD45 + immune cells, whereas samples 110 TP, 110 TC, 111 TP and 111 TC showed minimal immune presence (Fig. 7a).

These differences were maintained in the corresponding early passage PDOs (passage 1–2), as supported by the FACS analysis (Fig. 7b and Supplementary Fig. 6). t-SNE plots depict the distribution of immune (CD45 +, CD3 +, PD1 +) and epithelial (EpCAM +) cell populations in PDOs derived from the same four patients. Consistent with the tissue data, the PDOs derived from 112 TP, 112 TC and 117 TC retained a substantial immune fraction, including PD-1 + cells (respectively 43.8%, 39.5% and 28.9%), whereas the PDOs from 110 TP, 110 TC, 111 TP and 111 TC contained very few immune elements and showed nearly undetectable levels of PD-1 expression (respectively 0.9%, 1.8%, 7.69% and 6.7%) (Fig. 7b).

These findings further highlight the distinct immune profiles of the four samples and suggest a differential responsiveness to immunotherapeutic interventions.

Platinum-based chemotherapy remains the standard first-line treatment for BLC, while immunotherapy with PD-1/PD-L1 inhibitors is typically reserved for second-line administration after chemotherapy failure. However, emerging evidence suggests that combining chemotherapy with Nivolumab (a PD-1 inhibitor) may improve outcomes in patients with highly immune-infiltrated tumors [5]. To investigate this potential synergy, the response of early passage PDOs to Nivolumab, both as a monotherapy and in combination with chemotherapy (CDDP and GEM), was evaluated. To assess the functional relevance of immune infiltration, PDOs derived from BLC110 TP, BLC110 TC, BLC111TP, BLC111 TC, BLC112 TP, BLC112 TC and BLC117 TC were treated with chemotherapy (Combo: CDDP 10 μM + GEM 140 μM), Nivolumab (NIV 20–40 μg/mL), and their combination (Combo + NIV 20 μg/mL, Combo + NIV 40 μg/mL) (Fig. 7c). To ensure clinical relevance, Supplementary Table 6 compares the in vitro concentrations with reported maximum concentration (Cmax) values for CDDP [21], GEM [22] and Nivolumab [23, 24]. The 10 µM CDDP + 140 µM GEM dose reflects the upper clinical range, selected to apply maximal cytotoxic pressure while remaining translationally relevant. Nivolumab was tested at 20 and 40 µg/mL to reflect clinical plasma levels and assess dose-dependent immunomodulatory effects. The heatmap in Fig. 7c shows PDOs viability after 72 h of treatment. Notably, PDOs with higher immune infiltration, such as BLC112 TP, BLC112 TC and BLC117 TC, exhibited a significant reduction in cell viability when treated with the combination of Nivolumab and chemotherapy compared to chemotherapy alone, indicating an enhanced cytotoxic effect in samples with a robust immune microenvironment (Fig. 7c). Conversely, BLC110 TP and TC, which displayed low immune infiltration, showed no significant difference in viability between adopting chemotherapy alone and combination treatment. Similarly, BLC111 TP and TC, although exhibiting substantial cell death in response to chemotherapy, did not demonstrate further viability reduction with the addition of Nivolumab. More importantly, Nivolumab monotherapy did not affect cell viability in any of the samples, suggesting that its efficacy depends on its combination with chemotherapy, particularly in PDOs with higher immune infiltration (Fig. 7c).

These findings were further supported by the correlation analysis between PD-1 expression in PDOs and their response to treatments (Fig. 7d). A clear trend emerged, showing that samples with higher PD-1 + infiltration exhibited a substantially greater reduction in viability when treated with the combination therapy compared to chemotherapy alone—specifically, BLC112 TP, BLC112 TC and BLC117 TC showed reductions of approximately 45%, 61% and 68%, respectively, in cell viability with adding Nivolumab. In contrast, samples with low immune infiltration, such as BLC110 TC and BLC111 TC, did not display a similarly pronounced difference in viability between chemotherapy alone and the combined treatment, demonstrating that Nivolumab is ineffective in these cases.

Collectively, these results indicate that early passage PDOs can transiently preserve key features of the tumor immune microenvironment. The consistent immunotherapy responses observed in PDOs derived from both TC and TP samples suggest limited spatial variability in immune content, despite underlying tumor heterogeneity. This supports the robustness of PDOs in retaining immune-relevant features and highlights their potential for personalized immunotherapy testing.

## Discussion

BLC is a common malignancy with high recurrence rates and variable treatment responses [2, 4]. Despite therapeutic advances, outcomes often differ even among patients with similar clinicopathological features, largely due to inter-patient molecular heterogeneity. Understanding and functionally characterizing these variations is essential for improving patient specific treatment strategies and ultimately enhancing survival [4]. In recent years, PDOs have emerged as powerful preclinical models that reliably retain tumor specific characteristics, including histopathology, genomic alterations, and drug response patterns [6, 25]. In this study, by integrating data from two complementary European cohorts, Rome (IRE) and Bern (UniBE), a comprehensive overview of BLC heterogeneity was provided through the combined use of PDOs and ex vivo tissue slices, supported by high-dimensional FACS phenotyping.

While the UniBE cohort enabled multiparametric profiling and functional drug testing on native tissue slices, the IRE cohort allowed for the in-depth generation, molecular validation, and pharmacological testing of PDOs. PDOs generated from IRE specimens demonstrated strong histological and molecular fidelity to their parental tumors, and consistent with prior studies, most PDOs recapitulated the transcriptional and mutational features of their corresponding tissues [6, 7]. To explore the spatial complexity of BLC, PDOs were established from TC and TP regions in six patients who underwent RC. While RNA-seq-analysis demonstrated transcriptional similarity between matched tissues and PDOs, WES identified several region specific mutational differences, including allelic imbalances and variation in TMB, supporting the need for region resolved profiling to fully account for intra tumoral heterogeneity. Interestingly, these molecular variations had direct functional relevance: TC and TP derived PDOs exhibited distinct responses to CDDP, GEM, and to the FGFR inhibitor, erdafitinib. Consistent with previous studies, FGFR alterations, particularly FGFR3, are widely present in IRE BLC sub-cohort, detected in 6 of 12 patients [2, 26]. Erdafitinib, an FDA-approved FGFR inhibitor, has shown efficacy in FGFR2/3-altered BLC [27, 28]. In BLC17, both regions contained the FGFR3 p.Y375C mutation at similar VAFs, yet showed divergent drug sensitivities, suggesting the influence of either local signaling environments or microenvironment derived cues. Further, BLC15 PDOs bearing FGFR1 amplification failed to respond to erdafitinib, aligning with prior reports demonstrating limited effectiveness of FGFR inhibitors in tumors with FGFR1–4 amplification [29].

Recent advances in immune checkpoint inhibitors, including PD-1 and PD-L1 inhibitors, have shown promising results in clinical trials, improving overall survival and response rates [3, 6]. The combination of immunotherapy with chemotherapy and targeted therapies is being explored to enhance clinical outcomes and overcome resistance [6]. A central obstacle in advancing immunotherapy for BLC is the difficulty of modeling the tumor immune microenvironment in vitro. Here, multiparametric FACS phenotyping revelaed key immune and epithelial cell states in native UniBe tissues and early passage IRE PDOs. Flow cytometry of freshly resected UniBe specimens revealed heterogeneous cellular composition, distinguishing NMIBC from MIBC samples across epithelial, stromal, endothelial, and immune compartments. These data enabled epithelial landscape mapping, revealing striking heterogeneity, particularly in MIBC samples, where basal-like, EGFR-high populations were enriched. Importantly, FACS based deconvolution of early passage PDOs showed that PDOs transiently preserve key immune populations, including CD45 +, CD3 +, and PD 1 + cells, in proportions that mirror those of the original tissue. This finding was crucial, as it enabled functional assessment of immunotherapy responsiveness in PDOs, a capability rarely achievable in organoid systems due to rapid immune cell attrition. In immune infiltrated PDOs (e.g., BLC112), the combination of Nivolumab and CDDP–GEM markedly enhanced cytotoxicity compared to chemotherapy alone, whereas immune-cold PDOs derived from patients, such as BLC110 and BLC111, showed no additional benefit. These data parallel recent clinical findings showing superior responses to chemo immunotherapy in immune inflamed tumors [5] and support the hypothesis that baseline PD-1 + content, quantified here by FACS, may serve as a more functional biomarker of immunotherapy sensitivity than bulk measurements such as PD-L1 expression or TMB. The lack of regional (TC vs. TP) differences in immunotherapy response further suggests that immunomodulatory therapies may be less influenced by spatial genomic heterogeneity than targeted therapies.

Beyond PDOs, this study established an ex vivo tissue culture system using UniBe specimens that preserves full architectural and multicellular complexity. Untreated slices faithfully preserved the cellular composition, histo-architecture patterns of parental tissue, validating the fidelity of this model for short-term pharmacotyping studies. Across chemotherapy, receptor tyrosine kinase inhibition, and nivolumab-containing regimens, ex vivo profiling showed treatment-specific modulation of epithelial states. In particular, muscle-invasive samples exhibited a reduction of an EGFR-high, basal-like epithelial population after FGFR3 or EGFR blockade (erdafitinib, lapatinib, erlotinib), whereas chemotherapy tended to enrich basal-like features, findings that align with resistant phenotypes observed clinically and that were not as apparent in NMIBC, which remained comparatively stable in epithelial composition over acute pharmacological perturbations. Because slices conserve stromal and immune interactions that are progressively lost in culture, these ex vivo data provide mechanistic context for PDO-based viability results and strengthen confidence that observed sensitivities reflect clinically meaningful biology rather than artefacts of dissociation and expansion. The alignment between FACS defined epithelial clusters in tissues and their modulation in ex vivo slices underscores how high dimensional profiling can refine mechanistic interpretation of treatment responses.

Together, the convergence of PDO, ex vivo cultures and FACS based phenotyping findings constitutes a robust and clinically relevant framework for decoding the molecular and functional heterogeneity of BLC. The convergence of findings across platforms highlights the complementary strengths of each model: PDOs excel at capturing tumor intrinsic genomic and signaling dependencies, while ex vivo slices preserve tissue architecture and microenvironmental interactions that shape treatment responses but are typically lost in vitro. The addition of multiparametric FACS provides a high resolution lens for interpreting epithelial heterogeneity, stromal infiltration, and immune phenotypes, enabling more nuanced predictions of therapeutic outcomes. Collectively, these results reinforce the value of a dual platform approach for functional precision oncology in BLC and suggest that integrating PDO and tissue-slice based pharmacotyping with immune profiling may enable personalized therapy selection, improve immunotherapy stratification, and accelerate translational pipelines for targeted and combination therapies.

## Conclusion

This study demonstrates the translational value of BLC PDOs as models that preserve immune elements and capture functional diversity in drug responses, while also highlighting the complementary strengths of ex vivo tumor slice cultures in maintaining the full cellular architecture and microenvironment of the original tumors. By integrating spatial sampling, molecular and histopathological profiling, ex vivo pharmacological interrogation, PDO‑based drug testing, and high‑dimensional FACS‑based immune and epithelial analyses across two independent European cohorts, a comprehensive and multi‑scale overview of BLC complexity was provided. The incorporation of multiparametric FACS allowed us to map epithelial and immune heterogeneity with high resolution, revealing cellular states and immune infiltration patterns that were functionally recapitulated in early‑passage PDOs and modulated in ex vivo slices upon treatment. The concordance between PDO, FACS, and ex vivo findings underscores the importance of addressing both molecular heterogeneity and microenvironment‑driven functional heterogeneity when developing therapeutic strategies. Together, these platforms reinforce the value of PDOs and ex vivo slices as versatile, complementary tools in precision oncology, with substantial promise to guide individualized treatment selection, refine immunotherapy strategies, and enable clinically actionable functional precision medicine in BLC.

## Supplementary Information

Supplementary Material 1.

Supplementary Material 2.

Supplementary Material 3.

Supplementary Material 4.

Supplementary Material 5.

Supplementary Material 6.

Supplementary Material 7.

Supplementary Material 8.

## Data Availability

No datasets were generated or analysed during the current study.

## Abbreviations

BLC: Bladder Cancer
PDOs: Patient-derived organoids
TC: Central tumor
TP: Peripheral tumor
IRE: Regina Elena National Cancer Institute
UniBe: University of Bern
NMIBC: Non-muscle invasive bladder cancer
MIBC: Muscle invasive bladder cancer
TURBT: Transurethral resection of bladder tumor
RC: Radical cystectomy
PBMCs: Peripheral blood mononuclear cells
ULA: Ultra-low attachment
FFPE: Formalin fixed-paraffin embedded
IHC: Immunohistochemistry
ICC: Immunocytochemistry
WES: Whole exome sequencing
RNA-Seq: RNA- Sequencing
dPCR: Digital PCR
VEP: Variant effect predictor
PCGR: Personal cancer genome reporter
MAF: Minor allele frequence
LOH: Loss of heterozygosity
tSNE: T-distributed stochastic neighbor embedding
Ck: Cytokeratin
TCGA: The cancer genome atlas
TMB: Tumor mutational border
ssGSEA: Single sample Gene Set Enrichment Analysis
TME: Tumor microenvironment
SOC: Standard-of-care
CDDP: Cisplatin
GEM: Gemcitabine
FCM: Multiparametric flow cytometry
nMFI: Normalized mean fluorescence intensity
PT: Parental tumor
Cmax: Maximum concentration
