# Supplementary figures and images for: Bridging phenotype and function in bladder cancer using immuno-competent organoids and ex vivo drug screening
Source: J Exp Clin Cancer Res. 2026 Apr 1;45:117. doi: 10.1186/s13046-026-03701-x (PMC13173909; doi:10.1186/s13046-026-03701-x)

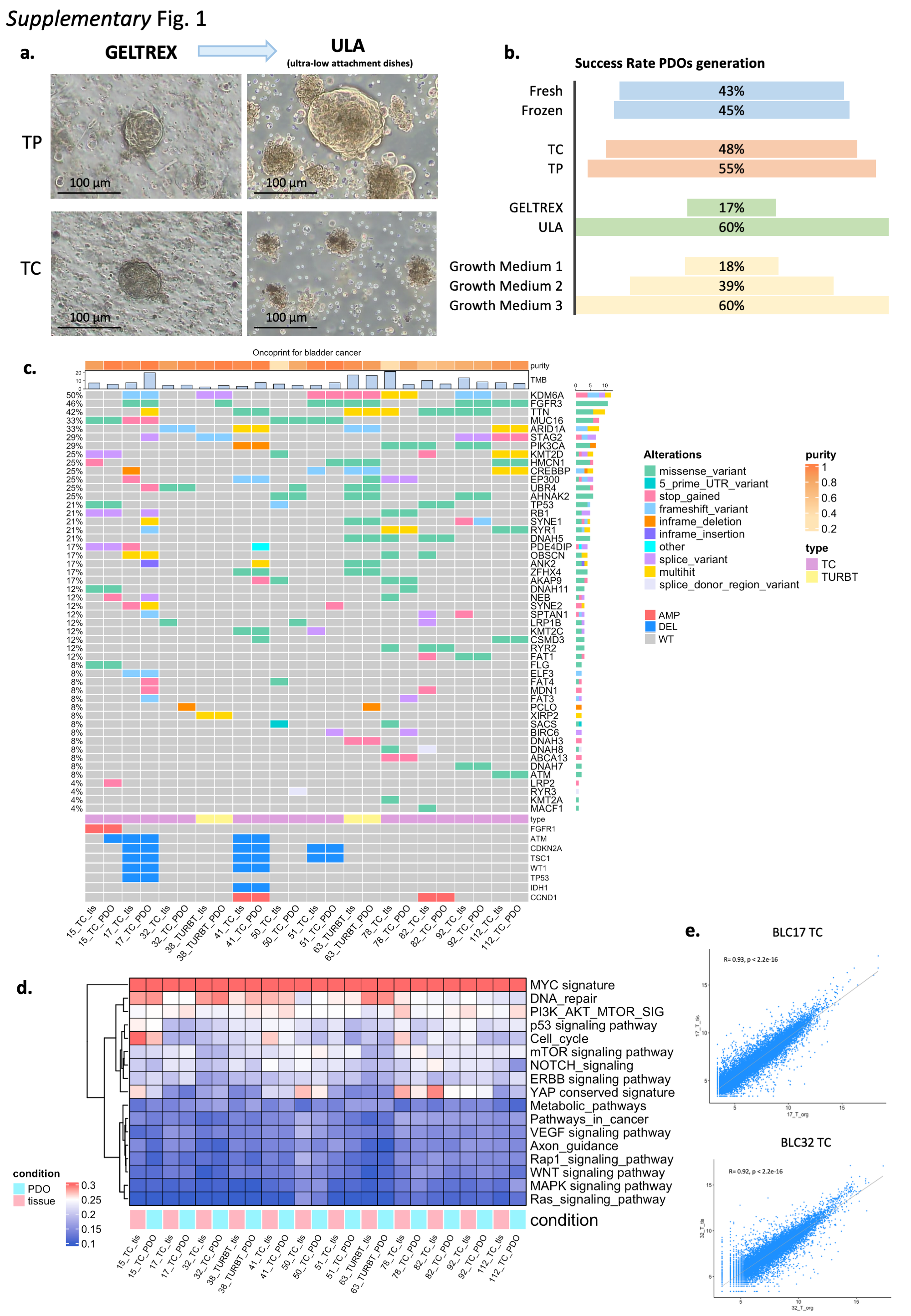

Supplement: Supplementary file 1 — Supplementary Material 1. [file 13046_2026_3701_MOESM1_ESM.tiff]

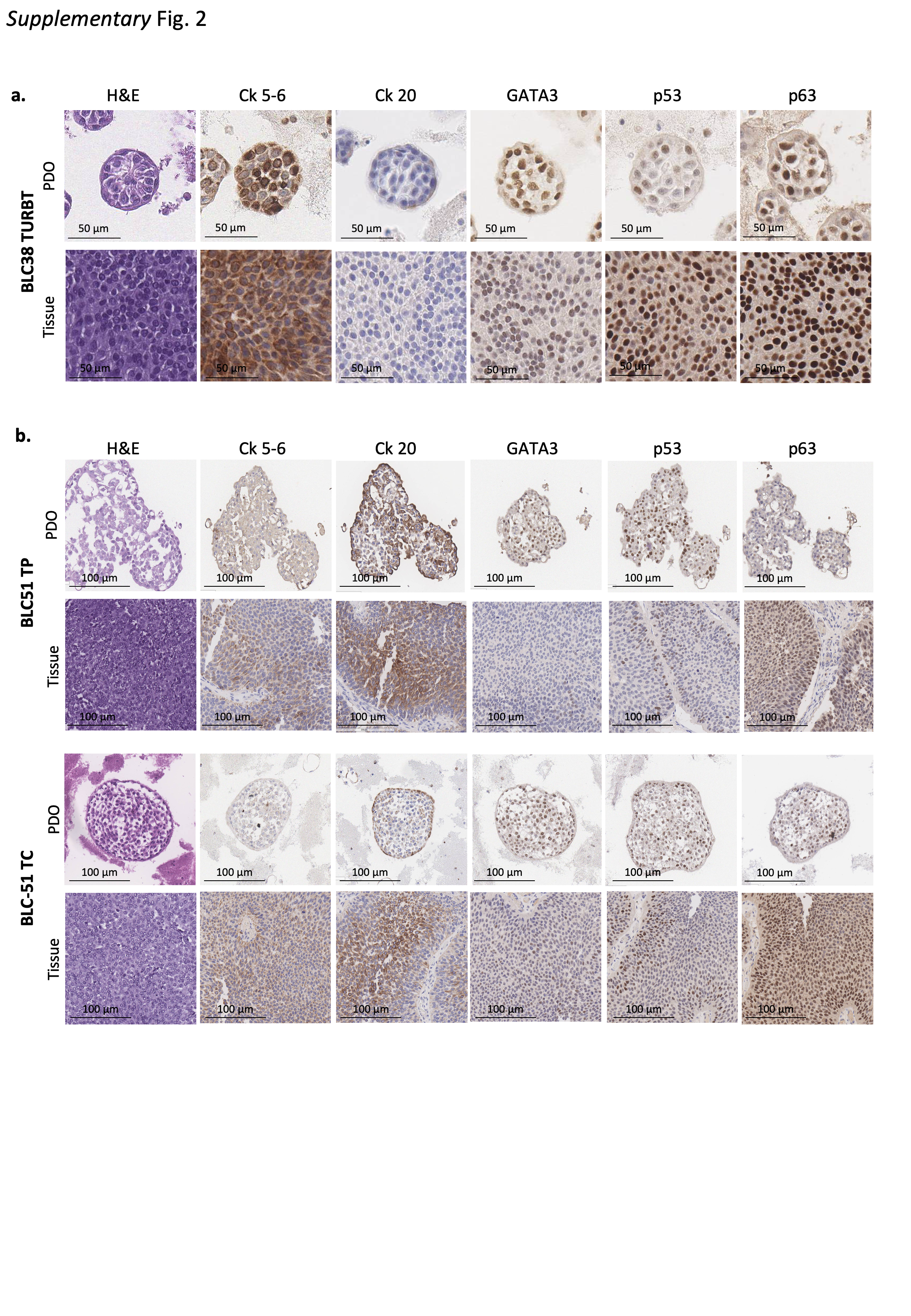

Supplement: Supplementary file 2 — Supplementary Material 2. [file 13046_2026_3701_MOESM2_ESM.tiff]

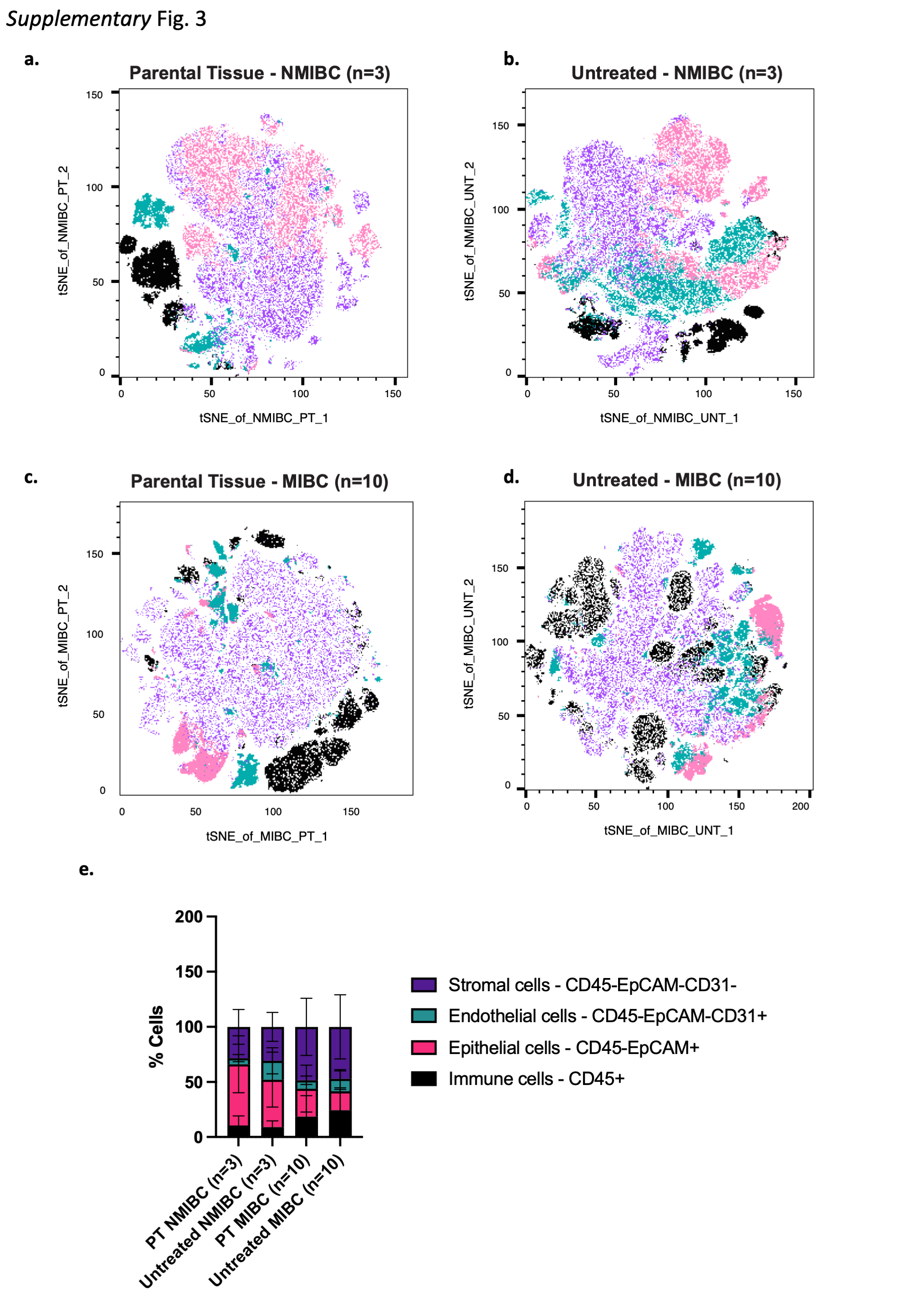

Supplement: Supplementary file 3 — Supplementary Material 3. [file 13046_2026_3701_MOESM3_ESM.tiff]

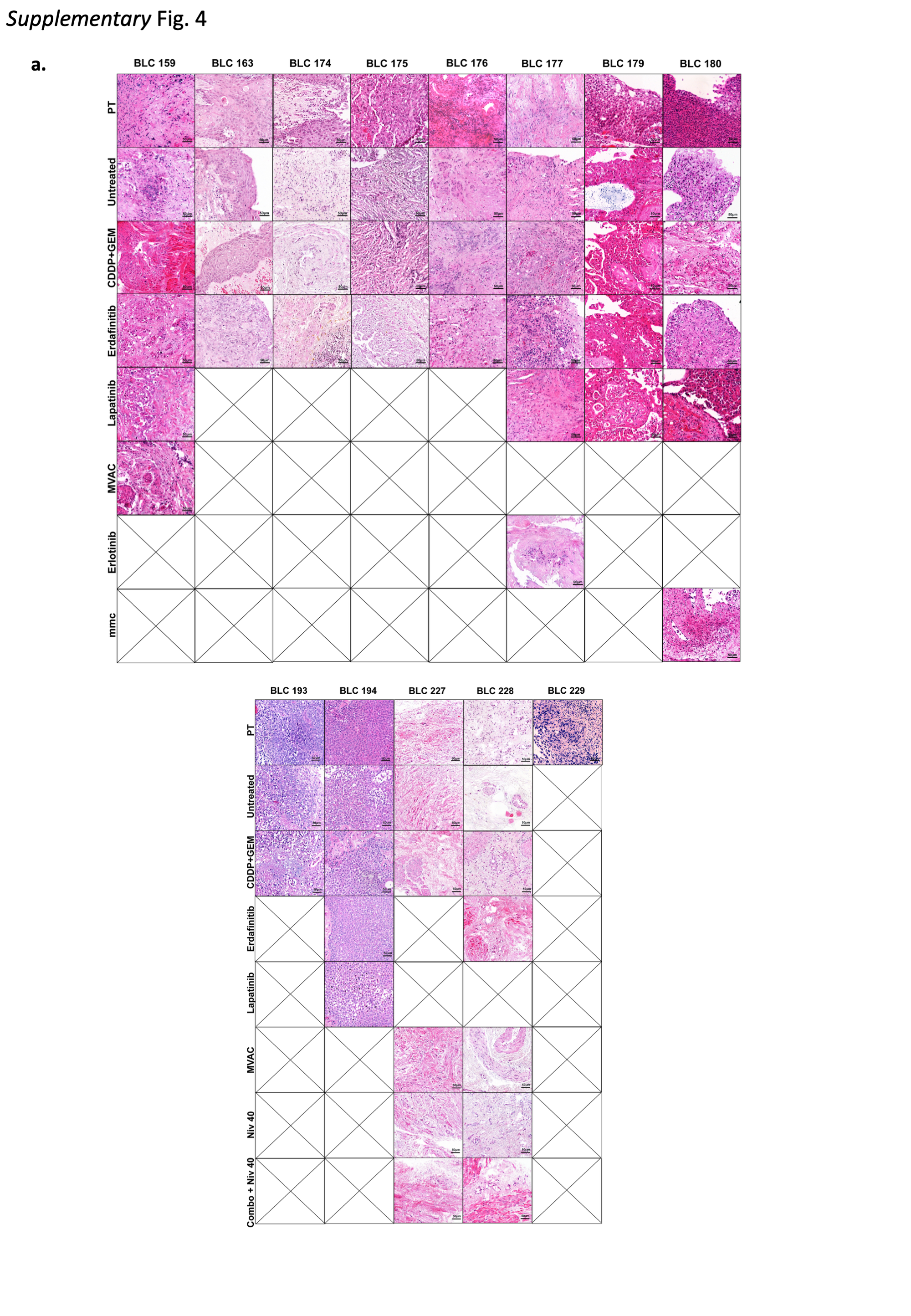

Supplement: Supplementary file 4 — Supplementary Material 4. [file 13046_2026_3701_MOESM4_ESM.tiff]

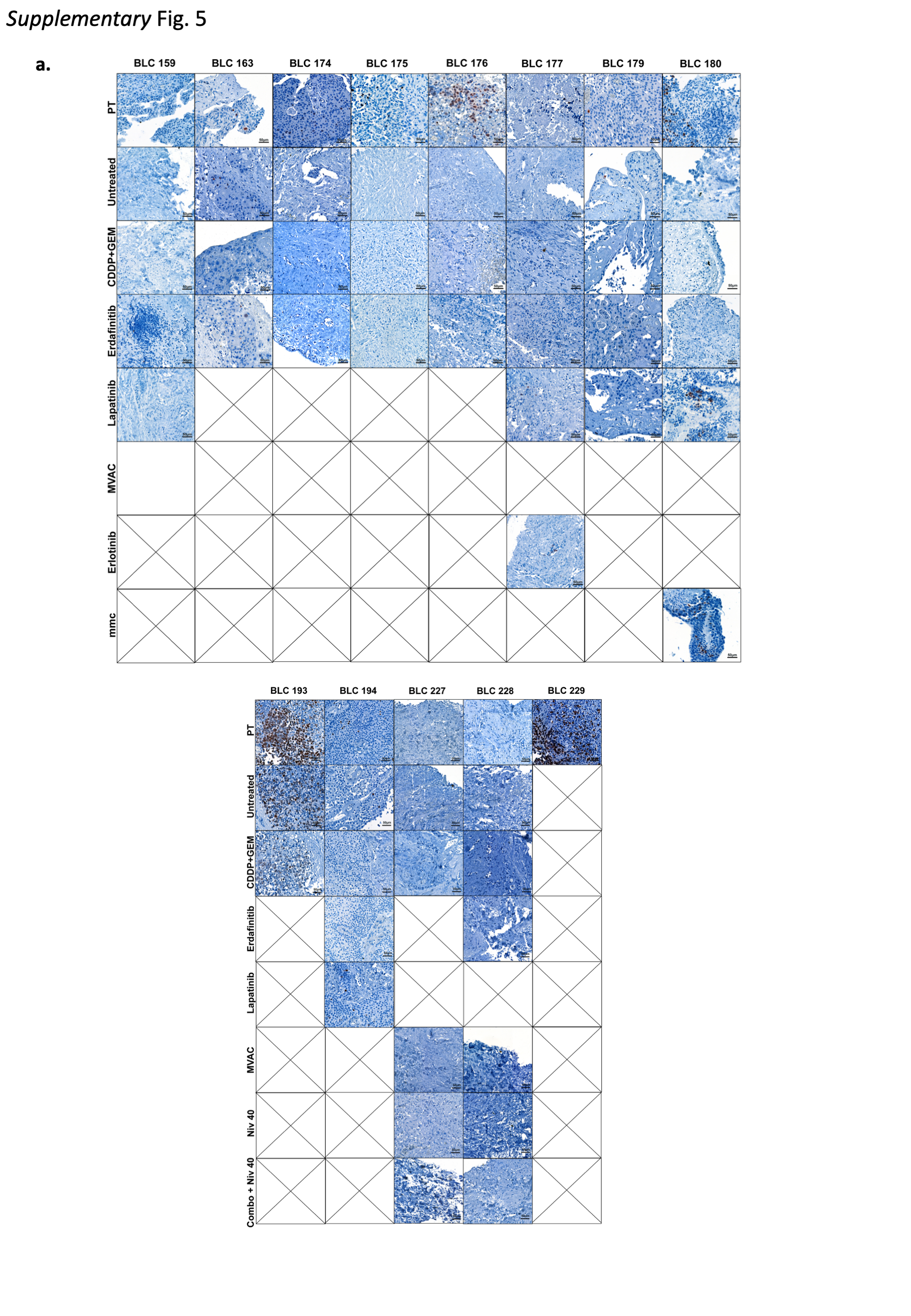

Supplement: Supplementary file 5 — Supplementary Material 5. [file 13046_2026_3701_MOESM5_ESM.tiff]

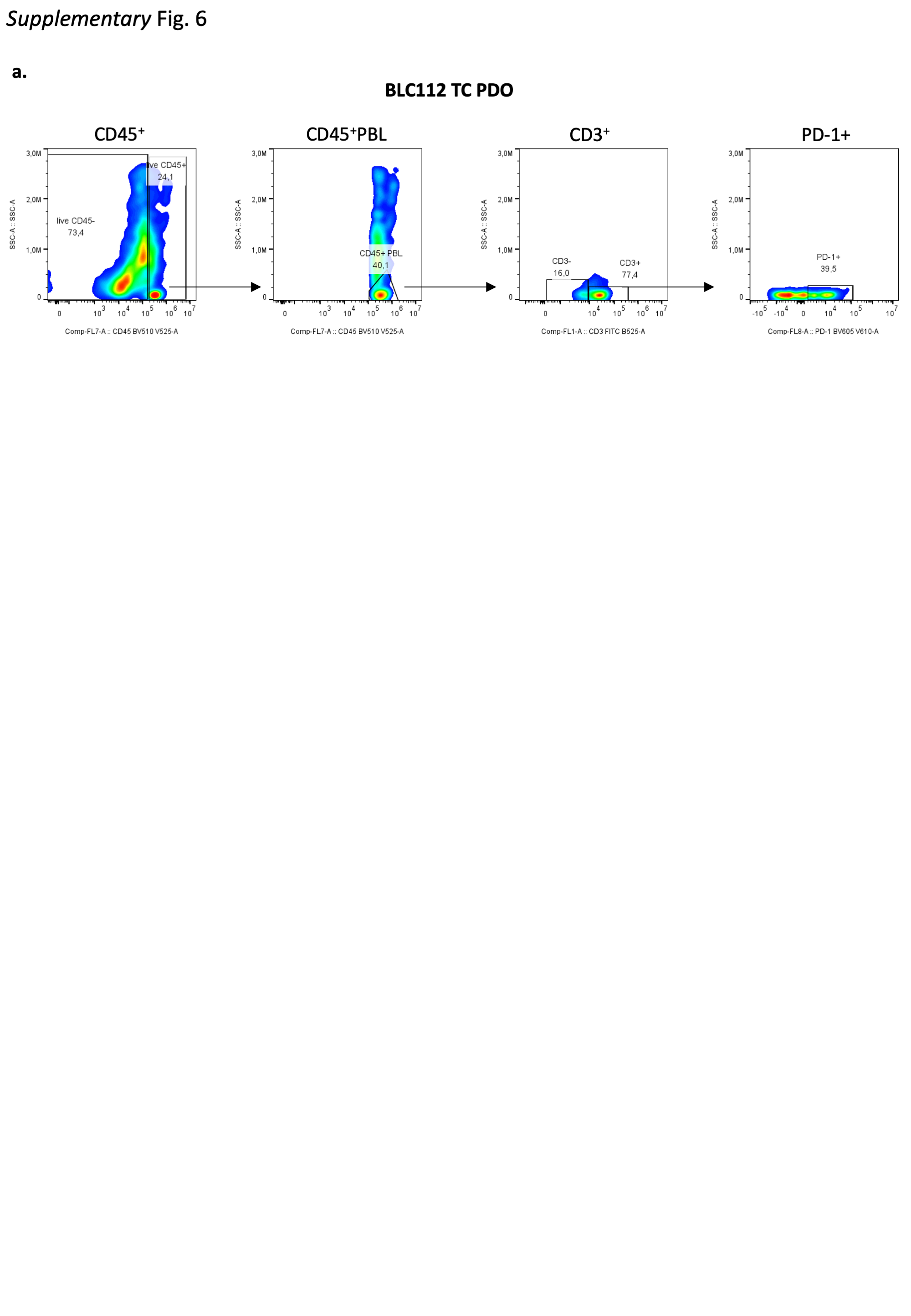

Supplement: Supplementary file 6 — Supplementary Material 6. [file 13046_2026_3701_MOESM6_ESM.tiff]
