# Supplementary material for: Bridging phenotype and function in bladder cancer using immuno-competent organoids and ex vivo drug screening
Source: J Exp Clin Cancer Res. 2026 Apr 1;45:117. doi: 10.1186/s13046-026-03701-x (PMC13173909; doi:10.1186/s13046-026-03701-x)
Supplement: Supplementary file 8 — Supplementary Material 8. [file 13046_2026_3701_MOESM8_ESM.docx]

**Supplementary Figure 1.** Establishment and characterization of bladder cancer PDOs from central tissues. **a.** Representative 5X magnification brightfields images of TP and TC PDOs growth in geltrex (left side) or in ultra-low attachment dishes (right side). **b.** Each bar represents the success rate of PDOs generation for different type of tissue (fresh/frozen, TC/TP) and different cultured conditions (geltrex/ULA, different growth medium). Growth Medium 1 (Mullenders J, et al., 2019), growth medium 2 (Minoli M, et al., 2023), growth medium 3 (new medium). ULA (Ultra-low attachment plates. **c.** Oncoplot of top mutated genes from WES analysis in matched TC tissues and PDOs samples. Tumor purity and TMB are reported on the top. **d.** Heatmap of cancer pathways expression in matched TC PDOs and tissues. **e.** Pearson’s correlation analysis of most highly expressed genes (~12.000) in BLC tissues and matched PDOs of two selected cases (BLC17 TC, BLC32 TC). BLC: bladder cancer; TP: peripheral tumor, TC: central tumor, PDO: patient-derived organoids.

**Supplementary Figure 2.** **a-b**. Comparative Haematoxylin and Eosin staining and immunohistochemistry staining for indicated markers of BLC organoids and matched tissues (BLC-38, BLC-51). BLC: bladder cancer; CK cytokeratin. TP: peripheral tumor, TC: central tumor.

**Supplementary Figure 3.** Main cellular composition of original parental tissue and untreated ex vivo slices. tSNE representing viable cells clustered as stromal, endothelial, epithelial, or immune cells in NMIBC (**a, b**) and MIBC (**c, d**), represented by experimental condition, parental tissue (**a, c**) and untreated sections (**b, d**). **e**. Fraction of viable cells from parental tissue and untreated sections in NMIBC and MIBC samples clustering as stromal, endothelial, epithelial, or immune cells. Gating strategy is described in the chart legend (**e**).

**Supplementary Figure 4.** **a.** Representative images of H&E stain on parental tissue and ex vivo culture conditions in 13 BLC samples. BLC: bladder cancer. Scale bar 50 μm.

**Supplementary Figure 5.** **a.** Representative images of Ki-67 stain on parental tissue and ex vivo culture conditions in 13 BLC samples. BLC229 ex vivo sections were not included for staining due to biological material limitation. Flow cytometry was prioritized over histological characterization. BLC: bladder cancer. Scale bar 50 μm.

**Supplementary Figure 6.** Flow cytometry of immune cell population of BLC112 TC PDO. **a.** The gating strategy and the percentage of positive cells are shown for a representative bladder cancer PDO. CD45+ cells were selected in live cells, then peripheral blood lymphocytes (PBLs) were identified and within them T lymphocytes as CD3+ cells. PD-1+ cells were gated in CD3+ T cells and expressed as percentage of positive cells.

**Supplementary Table 1.** The primary antibodies used for immunohistochemical analysis. RTU: Ready To Use

**Supplementary Table 2.** Antibodies used for flow cytometry analysis. 17 antibodies were used by the University of Bern, and 4 were used for IRE experiments

**Supplementary Table 3.** Tumor Mutational Burden (TMB) values of PDO from the IRE cohort obtained through Whole Exome Sequencing (WES) analysis.

**Supplementary Table 4.** Table showing Variant Allele Frequency (VAF%) and corresponding mutated residues across tumor tissues and matched PDOs.

**Supplementary Table 5** Table reporting CIBERSORT data from patient tissues and matched PDOs

**Supplementary Table 6.** List of plasmatic concentration of cisplatin, gemcitabine and nivolumab.
